# Squeezing More Juice out of Dielectric Elastomer Generators

**DOI:** 10.3389/frobt.2022.825148

**Published:** 2022-02-11

**Authors:** Samuel Rosset, Iain A. Anderson

**Affiliations:** Biomimetics Laboratory, Auckland Bioengineering Institute, University of Auckland, Auckland, New Zealand

**Keywords:** energy harvesting, soft generator, optimisation, analytical model, energy density, soft transducers, dielectric elastomers

## Abstract

Dielectric elastomer generators are soft structures capable of converting mechanical energy into electrical energy. Here, we develop a theoretical model of the triangular harvesting cycle that enables the harvesting of most of the available electrical energy while not requiring active monitoring of the charge-voltage state on the DEG. This cycle is therefore interesting for small-scale generators for which a monitoring circuit would be energetically too costly. Our model enables the identification of the optimal value of the circuit’s parameters such as storage capacitor and priming voltage values and show that for capacitance swings up to 6, 94% of the available electrical energy can be harvested. The model is experimentally validated with a conical generator, and the effect of non-constant deformation amplitudes is examined. Energy densities up to 46 mJcm^−3^ were obtained for an electric field of 50 V µm^−1^.

## Introduction

Dielectric elastomer transducers (DETs) consist of an elastomeric dielectric film sandwiched between a pair of compliant electrodes, thus forming a rubbery deformable capacitor. They can be used as actuators for soft machines, as sensors, or as generators (Pelrine et al., 2000; Anderson et al., 2012; Rosset and Shea, 2016; Moretti et al., 2020b). In generator mode, they transform mechanical energy into electrical energy. Their stretchability makes them particularly well-suited to harvest energy from large-amplitude, low frequency sources of mechanical energy. Dielectric elastomer generators (DEGs) have been proposed to harvest energy from ocean waves (Kornbluh et al., 2011; Jean et al., 2012; Moretti et al., 2020a), from human body motion (Kornbluh et al., 2011; Savage, 2012; Vu-Cong et al., 2013), or from the swaying motion of tree branches (Anderson et al., 2011). The low density and compliance of DEGs enable to make devices that can harvest energy unobtrusively and silently from the motion of limbs, such as the knee (Jean-Mistral et al., 2008), and the collected energy can be used to power sensors for physiological monitoring or for the gps tracking of large predators. Harvesting energy from tree branch motion could enable the deployment of wireless sensor networks for forest health or fire monitoring.

One key question remains to evaluate the feasibility of these applications: how much energy can realistically be harvested by DEGs, especially for miniaturised devices comprised of only a few grams of active material? Koh et al. have investigated the theoretical limits of DEGs, by considering their different failure modes (mechanical rupture of the elastomer, electrical breakdown of the elastomer, electromechanical instability and loss of tension). They showed that an energy density of more than 1 J g^−1^ could be harvested per cycle (1.7 J g^−1^ when the acrylic elastomer VHB from 3M is used as dielectric, and 1.3 J g^−1^ for natural rubber) (Koh et al., 2011). Assuming a low harvesting frequency of 1 Hz (e.g. walking frequency), the theoretical harvestable power density is in the order of 1 Wg^−1^. Experimentally, Shian et al. have obtained an energy density of 0.76 J g^−1^ (Shian et al., 2014), which is not too far from the absolute maximum predicted by Koh’s model. However, these values are obtained by pushing DEGs to their limits in a laboratory setting. In order to provide a useful lifetime, any practical application would have to stay well away from the failure modes that otherwise limit the feasible space of DEGs. They are thus expected to harvest only a fraction of the energy density values mentioned previously.

Designing DEGs for long-term operation has implications on both mechanical and electrical parameters. The cyclic mechanical deformation needs to be chosen far from the rupture limit of the elastomer for two reasons: 1) to avoid fatigue of the dielectric membrane (Bruch et al., 2020), and 2) to avoid degradation of the compliant electrodes (Rosset et al., 2017; de Saint-Aubin et al., 2018). In addition, the electric field in the structure must be limited to a value well below the dielectric breakdown limit of the material to ensure long-term operation, as parameters such as humidity can influence the breakdown field (Fasolt et al., 2019; Albuquerque and Shea, 2020). As described in more detail in “*The energy harvesting cycle*” section, combining these two effects leads to a harvestable energy density of the order of 0.1 J g^−1^ for a DEG using silicone elastomer as dielectric, i.e. about ten times lower than the maximum energy density that would be available by pushing the DEG to its limit. Furthermore, while large kW-range DEGs can invest some of their energy output to monitor their deformation and ensure that the electronic harvesting circuit is operating at its optimal point, miniaturised DEGs usually rely on simple electronic harvesting circuits operating without feedback. These circuits are tuned for a precise amplitude of deformation. However, except in a lab testing environment, a natural source of mechanical energy for the deformation of a DEG such as walking is stochastic and will see its amplitude change over time. In this contribution, we investigate the ideal electronic circuit topology to harvest energy from a miniaturised DEG and optimise its parameters to maximise the energy harvested from a source of mechanical energy with varying amplitude and frequency.

## The Energy Harvesting Cycle

**FIGURE 1:**
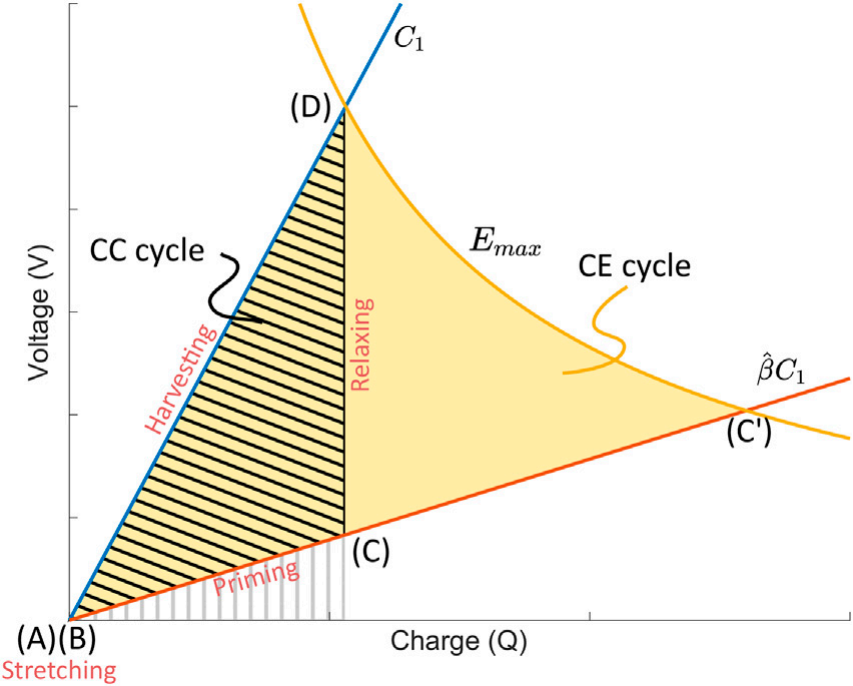
Constant charge (CC) and Constant field (CE) harvesting cycle in the Q-V plane of a DEG which is stretched from a capacitance *C*_1_ to $\hat{\beta }C_{1}$ and relaxed back to *C*_1_. The grey hatched area with vertical stripes represents the amount of input energy required to prime the generator for the CC cycle. In contrast, the black hatched area represents the net energy gain per cycle. The yellow area represents the net energy gain per cycle in the case of a constant field (CE) cycle, for a field value equal to the maximal electric field *E*_max_.

The working principle of a DEG has been detailed in the literature [e.g. Moretti et al. (2020b)], and is illustrated in the charge-voltage (Q-V) plane in Figure 1. Briefly, an uncharged DEG with a capacitance *C*_1_ in its relaxed state is mechanically deformed until it reaches its maximal capacitance of $\hat{\beta }C_{1}$ [segment (A)–(B)]. As this is a purely mechanical action, nothing takes place on the Q-V plane. *β* (*t*) = *C* (*t*)/*C*_1_ is a periodic function that describes the **capacitance swing** of the DEG, taking values between 1 and $\hat{\beta }$ . The maximum capacitance swing $\hat{\beta }$ is one of the key factors influencing the amount of collected energy. A priming charge is then loaded on the DEG [segment (B)–(C)], with the area under the segment (hatched with grey vertical line) representing the amount of electrical energy required to prime the DEG. The DEG is then relaxed, and its capacitance decreases back to *C*_1_ [segment (C)-(D)]. For the cycle shown here, we consider that the DEG is completely disconnected from any circuit for this phase, meaning that—neglecting leakage—the charge on the DEG remains constant. Consequently, the segment (C)-(D) is vertical, with the voltage at point (D) being higher than the voltage at point (C). Finally, the electrical charges are transferred to a harvesting circuit [segment (D)-(A)]. The gross energy collected is represented by the area below segment (D)-(A), making the net energy collected per cycle equal to that of the hatched triangle (AB-C-D).

We define a maximum electric field *E*_max_ that must never be exceeded at any point of the cycle. It is chosen low enough to prevent dielectric breakdown of the elastomer membrane even for long-term operation and ensure that electromechanical instabilities do not occur during the cycle. It is typically set between 70 and 80% of the breakdown field of the material, and therefore represents a *nominated* maximal field that we never want to exceed. The yellow line in Figure 1 describes the points at which the electric field in the DEG is equal to the critical nominated value *E*_max_ (c.f. Supplementary Material for a derivation of the equation of this line). Points on the left of the curve are at a lower electric field than *E*_max_, and points on the right are at a higher electric field, and define a region of the Q-V plane that the harvesting cycle must not enter. It can be seen that the constant charge (CC) cycle shown in Figure 1 has been designed to reach *E*_max_ a point (D), and therefore represents the optimal CC cycle achievable for the chosen values of *E*_max_ and $\hat{\beta }$ .

Figure 1 also shows that if we prime the DEG to a higher voltage until the maximal field is reached (point C′), and then keep the field constant at a value *E*_max_ during the relaxation, the energy gain per cycle, given by the yellow area, would be considerably higher than the constant charge case. This is the constant electric field (CE) cycle, whose energy density per cycle is given by Moretti et al. (2020b):

$$w_{CE}=\varepsilon E_{max}^{2}\ln\sqrt{\hat{\beta }}.$$ (1)

A shoe-heel stacked DEG (Savage, 2012; McKay et al., 2015) would exhibit a capacitance swing of $\hat{\beta }=4$ if compressed by half of its initial height (an appreciable deformation for a heel). If made of silicone (*ɛ* = 2.8 × 8.85 ⋅ 10^–12^ Fm^−1^) and operated at *E*_max_ = 80V µm^−1^, a CE cycle would therefore lead to an energy density of 0.11 Jcm^−3^, which would represent the maximal amount of harvestable energy for this nominated maximal field and capacitance swing. The CC cycle only harvests a fraction of this available energy, so the CE cycle should be preferred. However, it is more difficult to implement, as it requires active control of the charge and voltage of the DEG during the discharge phase, which calls for complex bidirectional high-voltage power supplies (Eitzen et al., 2011; Todorcevic et al., 2013) and monitoring of the capacitance and voltage level on the DEG. Although not a problem for large scale generators such as wave energy converters, smaller devices usually rely on simple harvesting circuits comprising a limited amount of components (Kaltseis et al., 2014; McKay et al., 2015; Huang et al., 2013), thus making the use of a CE cycle impractical.

**FIGURE 2:**
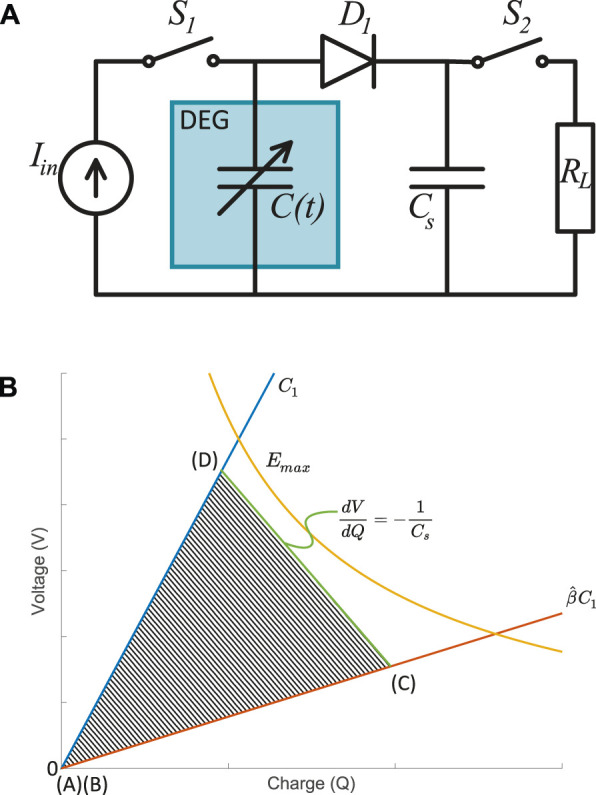
Triangular harvesting cycle. **(A)** The electronic circuit around the DEG consists of a power supply, 2 switches, a diode, and a storage capacitor *C*_*s*_. The Resistance *R*_*L*_ represents a load or a harvesting circuit to which the charges are transferred at the end of each cycle through the switch *S*_2_. **(B)** Triangular cycle in the Q-V plane. The slope of the segment (C)-(D) is controlled by the value of the storage capacitor.

In 2014, Shian et al. introduced a triangular cycle based on a simple electronic circuit (Figure 2A) that enables to cover a larger area of the feasible region (Shian et al., 2014). The idea consists in placing a storage capacitor of value *C*_*s*_ in parallel with the DEG and separated by a diode. One switch (*S*_1_) controls the charging, while a second switch (*S*_2_) enables to discharge the generator (here through the load *R*_*L*_, but in practice into a step-down circuit). A discharged DEG is stretched to $\hat{\beta }$ [(A)-(B)]. With switch *S*_2_ open, switch *S*_1_ is closed to prime the DEG. Once point (C) is reached, switch *S*_1_ is open and the DEG is relaxed. During this phase, charges are transferred from the DEG (whose capacitance decreases) to *C*_*s*_ through diode *D*_1_, causing a decrease of charges on the DEG and an increase of its voltage (Figure 2B). The slope of segment (C)-(D) is equal to −1/*C*_*s*_ (c.f. Supplementary Material). Therefore, the value of the storage capacitor *C*_*s*_ and of the charging voltage (voltage at point C) can be chosen so that segment (C)-(D) becomes tangent with the maximal electric field line. The triangular cycle therefore approaches the quantity of harvested energy that would be available with a constant field cycle. The concept of triangular cycles has been implemented in DEGs, for example by Moretti et al. (2017), Moretti et al. (2018) with circular diaphragm DEGs. However, no model exists to maximise and predict the energy that can be extracted.

In this contribution, we investigate the optimal charging voltage and storage capacitor required to maximise the generated energy per cycle of the triangular harvesting cycle. We analyse the impact of a non-constant deformation amplitude on the harvesting performance. Because the CE cycle represents the maximal amount of energy that can be harvested given our requirements (keeping the electric field below *E*_max_ and for the chosen value of capacitance swing), we will use it as a reference metric against which to normalise the output of other cycles.

## The Triangular Cycle

### The Optimal Case

We consider a DEG which is periodically stretched with a capacitance swing $1\leq \beta \left(t\right)\leq \hat{\beta }$ . The capacitance swing is related to the deformation of the DEG, but the exact relation between the stretch ratio and *β* depends on the topology of the generator (Moretti et al., 2020b). Furthermore, the stretch ratio of the DEG is, in turn, related to the input mechanical energy with a relation that depends on the stiffness of the actuator and impedance matching. When *β* = 1, the DEG has a capacitance $C_{1}=\varepsilon S_{1}^{2}/\Omega$ , with *ɛ* the permittivity of the elastomer, *S*_1_ the surface of the DEG capacitor, and Ω the volume of elastomer. We use index 1 for the capacitance and surface in the minimum capacitance configuration to indicate that a prestretch of the device may be present (with index 0 representing the device without any internal stress). As we consider the elastomer to be incompressible, Ω remains constant irrespective of the stretch state of the device.

**FIGURE 3:**
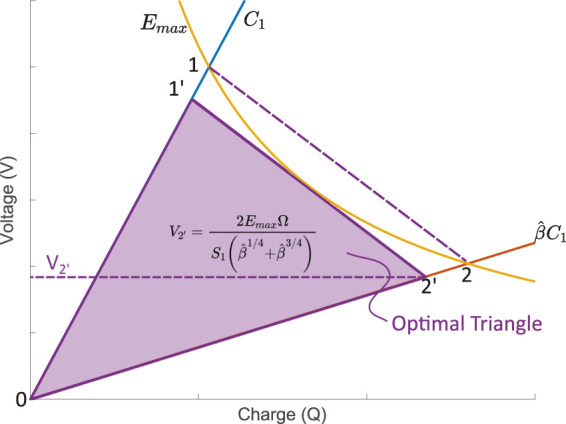
The optimal (OT) cycle is defined using a line linking the intersection of the maximal field line with the *C*_1_ isocapacitance line (point 1), and with the $\hat{\beta }C_{1}$ isocapacitance line (point 2). This line is then shifted until it becomes tangent with the maximal electric field limit to form line 1′-2’. The purple rectangle shows the net energy produced for each cycle.

We define the optimal triangle (OT) cycle as follows (Figure 3): we impose the slope of the relaxation process as that of the line that connect the intersection of the maximal field line with the isocapacitance line *C*_1_ (point 1) to the intersection of the maximal field line with the isocapacitance line $\hat{\beta }C_{1}$ (point 2). This line has a slope equal to $-1/C_{1}\sqrt{\hat{\beta }}$ (c.f. Supplementary Material), which means that the capacitance of the storage capacitor must be chosen to be:

$$C_{s}=C_{1}\sqrt{\hat{\beta }}.$$ (2)

To ensure that the electric field within the DEG remains below the maximal value *E*_max_ during the complete harvesting cycle, segment 1−2 is translated until it becomes tangent with the maximal field line, thus forming segment 1′−2’. The charge and voltage state of the DEG at points 1′ and 2′ are derived in the Supplementary Material and summarised in Table 1. The value of *V*_2’_ defines the priming voltage required to optimise the cycle. Therefore the two key parameters to optimise the triangle cycle is the choice of the storage capacitor, given by Eq. (2), as well as the voltage at which the switch *S*_1_ must be opened to stop the charging process, given by:

$$V_{2^{\prime }}=\frac{2E_{max}\Omega }{S_{1}\left({\hat{\beta }}^{1/4}+{\hat{\beta }}^{3/4}\right)}.$$ (3)

**TABLE 1:** Expression of the charge and voltage of the DEG capacitor at point 1′ and at point 2’.

	Q	V
Point 1′	$Q_{1^{\prime }}=\frac{2\varepsilon E_{max}S_{1}}{{\hat{\beta }}^{1/4}+{\hat{\beta }}^{-1/4}}$	$V_{1^{\prime }}=\frac{2E_{max}\Omega }{S_{1}\left({\hat{\beta }}^{1/4}+{\hat{\beta }}^{-1/4}\right)}$
Point 2′	$Q_{2^{\prime }}=\frac{2\varepsilon E_{max}S_{1}}{{\hat{\beta }}^{-3/4}+{\hat{\beta }}^{-1/4}}$	$V_{2^{\prime }}=\frac{2E_{max}\Omega }{S_{1}\left({\hat{\beta }}^{1/4}+{\hat{\beta }}^{3/4}\right)}$

The energy generated per cycle, equal to the area of the purple triangle on Figure 3, can be calculated as the energy extracted from the generator at the end of the cycle, minus the energy injected in the generator during priming. The energy required to prime the generator (and the storage capacitor that is connected in parallel) is equal to:

$$W_{in}=\frac{1}{2}\left(\hat{\beta }C_{1}+C_{s}\right)V_{2^{\prime }}^{2}=\frac{C_{1}}{2}\left(\hat{\beta }+\sqrt{\hat{\beta }}\right)V_{2^{\prime }}^{2},$$ (4)

where we have replaced the value of the storage capacitor by it is optimal value (Eq. (2)). After relaxation, when switch *S*_2_ is closed, the energy stored in the DEG and the storage capacitor is harvested, and the amount of collected energy is:

$$W_{out}=\frac{1}{2}\left(C_{1}+C_{s}\right)V_{1^{\prime }}^{2}=\frac{C_{1}}{2}\left(1+\sqrt{\hat{\beta }}\right)V_{1^{\prime }}^{2}$$ (5)

The values of the voltage at points 1′ and 2′ are given in Table 1. The net energy gain per cycle is obtained by subtracting Eq. 4 from Eq. 5. Dividing by the volume of the elastomer Ω leads to the net energy density produced per cycle for an optimal triangle (OT) cycle.

$$w_{OT}=2\varepsilon E_{max}^{2}\frac{\sqrt{\hat{\beta }}-1}{\sqrt{\hat{\beta }}+1}.$$ (6)

This value can be normalised by the the dielectric permittivity of the elastomer and the square of the maximum field imposed to the system to have a metric that only depends on the capacitance swing.

$$\frac{w_{OT}}{\varepsilon E_{max}^{2}}=2\frac{\sqrt{\hat{\beta }}-1}{\sqrt{\hat{\beta }}+1}.$$ (7)

**FIGURE 4:**
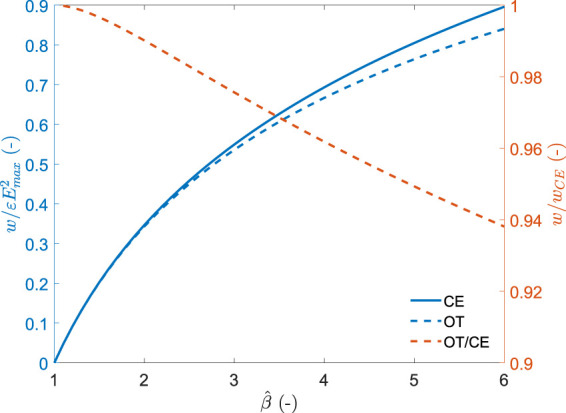
normalised energy density harvested per cycle for the optimal triangle (OT) and constant electric field (CE) cycles as a function of the capacitance swing (blue). Relative performance of the OT cycle with respect to the CE cycle (red).

The normalised energy density as a function of the peak capacitance swing is shown in Figure 4 and compared to the constant field (CE) cycle given by Eq. (1). For the range of capacitance swing depicted in the figure, the optimal triangle cycle leads to very close performance to that of the constant field cycle. Harvested energy densities are more than 93.8% of that of the CE cycle. The absolute value of harvested energy density depends on the material permittivity and the square of the maximal field *E*_max_. Values of $\varepsilon E_{max}^{2}$ for DEGs typically lie between 0.1 Jcm^−3^ and 2.2J cm^−3^ (Moretti et al., 2020b). The optimal triangle cycle enables to harvest a quantity of energy that is very close to the total amount of harvestable energy, using a simple electronic circuit requiring a few passive components and two switches. However, the values shown in Figure 4 can only be obtained if the storage capacitor is chosen to match the deformation, and if the charging voltage is adapted to bring the relaxation curve to be tangent to the maximal field line. The dependence of the storage capacitor value to the capacitance swing [Eq. (2)] imposes limitations on the flexibility of the circuit to adapt to varying amplitude of deformation. The demonstration that the OT cycle as defined here represents the largest amount of collectable energy is detailed in section 3.5 of the Supplementary Material.

### Non-optimal Storage Capacitor

**FIGURE 5:**
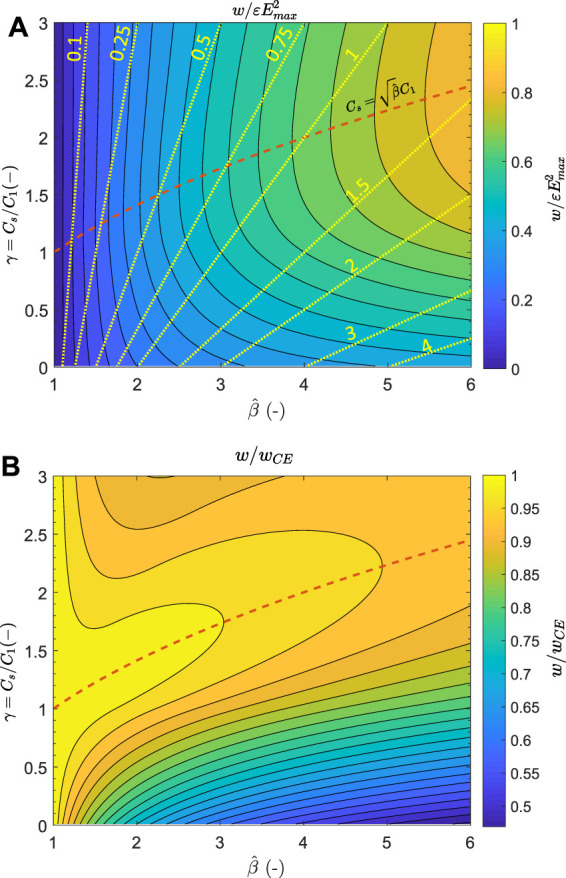
Normalised harvested energy density **(A)** and harvested energy density relative to the constant electrical field cycle **(B)** as a function of the capacitance swing and value of the storage capacitor. The red dashed line represents the optimal value of *C*_*s*_, and the yellow lines represent the ratio of the harvested energy with respect to the priming energy.

Even when the amplitude of the DEG deformation is well known, generating an OT cycle is made difficult by the need for a precise value of capacitor *C*_*s*_. The voltage on a DEG can easily reach up to 5 kV or more, and the storage capacitor must be able to sustain the same voltage. High-voltage capacitors only exist in limited capacitance values and are expensive. Finding the exact value to match the capacitance swing according to Eq. (2) can be difficult. Therefore, we compute the normalised harvested energy density for different values of the storage capacitance to evaluate the impact of using a non-ideal value of the storage capacitor. We assume that the peak capacitance swing $\hat{\beta }$ is known, which enables us to adjust the charging voltage *V*_2’_ so that the maximal electric field *E*_max_ is reached during the relaxation process. The procedure, and the expression to calculate *V*_2’_ as a function of $\hat{\beta }$ and the value of the storage capacitor is given in the Supplementary Material, with the required optimal priming voltage given in Table 2. The resulting harvested energy density is shown in Figure 5. The value of the storage capacitor is described by the parameter *γ* = *C*_*s*_/*C*_1_, with the value of the optimal storage capacitor as described by Eq. (2) highlighted by the red dashed line.

**TABLE 2:** Optimal priming voltage as a function of the capacitance swing $\hat{\beta }$ , and the relative capacitance of the storage capacitor *γ* = *C*_*s*_/*C*_1_.

	*V*_2’_ (Priming voltage)
0 ≤ *γ* < 1	$\frac{\Omega E_{max}}{S_{1}}\frac{\gamma +1}{\gamma +\hat{\beta }}$
$1\leq \gamma \leq \hat{\beta }$	$\frac{\Omega E_{max}}{S_{1}}\frac{2\sqrt{\gamma }}{\hat{\beta }+\gamma }$
$\gamma >\hat{\beta }$	$\frac{\Omega E_{max}}{\sqrt{\hat{\beta }}S_{1}}$

Figure 5A shows the normalised harvested energy density, and Figure 5B shows the harvested energy density relative to that of a constant electric field (CE) cycle. For a fixed value of $\hat{\beta }$ , the graph shows that over-sizing the storage capacitor has little effect. In contrast, an undersized capacitor leads to substantial reduction in harvested energy. However, another metric to consider is the ratio of harvested energy with respect to the input electrical energy required to prime each cycle (Graf et al., 2011). This ratio is indicated by the yellow lines in Figure 5A, and shows that a smaller capacitance *C*_*s*_ increases the relative amount of harvested energy. The optimum is a trade-off between absolute and relative energy gains and depends on the efficiency of the extraction and step-down electronics. Indeed, after the DEG relaxation and resulting energy gain, the stored electrical energy must be extracted from the DEG, and most likely converted to a low voltage. Suppose the circuit performing these operations has a low efficiency, and the relative amount of generated energy compared to the required priming energy is low. In that case, the whole system could end up dissipating power instead of generating any.

The amount of harvested energy is shown not to be very sensitive to the value of the storage capacitor because the deviation of the capacitance value compared to the optimum can be partially compensated by adapting the charging voltage. This means that a harvesting circuit with a storage capacitor not exactly tailored to the capacitance swing of the generator is still capable of harvesting a large part of the available energy. However, the priming voltage *V*_2’_ must be adjusted accordingly using the equations of Table 2. These equations depend on both *γ* and $\hat{\beta }$ , and therefore assume knowledge of these two values. If the former can easily be measured, the latter assume a well-defined and constant amplitude of deformation. As discussed in the next section, this is usually not the case, and the impact of a varying deformation of the generator on the harvested energy needs to be evaluated.

## Varying Deformation Amplitude

For most environmental energy sources deforming a DEG, the capacitance swing $\hat{\beta }$ is not constant over a large number of cycles but follows a statistical distribution. This impacts the amount of harvested energy with the OT cycle, as the value of the harvested energy depends on that of the optimal storage capacitor, which, in turns, depends on the capacitance swing. Here, we will consider the case of a normal distribution of $\hat{\beta }$ , with a mean value *μ* and a standard deviation *σ*.

The two fundamental resulting questions are: Given a distribution of capacitance swing, for which particular value of $\hat{\beta }$ should the storage capacitor be chosen to maximise the amount of harvested energy? And what is the average energy density produced per cycle? To answer these questions, we will consider two harvesting circuit strategies: 1) A “set-and-forget” approach for which the circuit is optimised for a defined deformation amplitude and operated without adjustments, and 2) An adaptive approach in which the parameters of the circuit are adapted on the fly to the effective deformation amplitude.

### Set-And-Forget Harvesting Strategy

Fixing the parameters of the harvesting circuit (namely the value of the storage capacitor *C*_*s*_, and the priming voltage *V*_2’_) does not require measuring the effective deformation with external sensors or a self-sensing scheme (Rizzello et al., 2018). It can be argued that the harvesting circuit described in Figure 2 requires active measurement of the deformation for proper operation of the switches *S*_1_ and *S*_2_. However, if the priming source is limited to an output voltage *V*_2’_, switch *S*_1_ can be replaced by a passive diode. Switch *S*_2_, which controls the transfer of the charges to the step-down converter, can be replaced by a break over switch (Lo, 2015), thus making it possible to implement this simple harvesting circuit without the need for active measurement of the deformation of the generator. This is particularly interesting for small-scale harvesting systems designed to harvest milliwatts of power.

With this approach, we set a deformation set point ${\hat{\beta }}_{s}$ at which to tune the circuit. We use Equation 2 to calculate the value of the storage capacitor, and Equation 3 to calculate the priming voltage. If the actual deformation of the DEG is smaller than ${\hat{\beta }}_{s}$ , then the amount of generated energy will be lower than that of the OT cycle. If the deformation is larger, the electric field will exceed *E*_max_ during the relaxation phase and the set-and-forget method thus requires to physically limit the mechanical deformation of the harvester to ensure that $\hat{\beta }\leq {\hat{\beta }}_{s}$ at all time. The detailed equations of this approach are given in the Supplementary Material.

**FIGURE 6:**
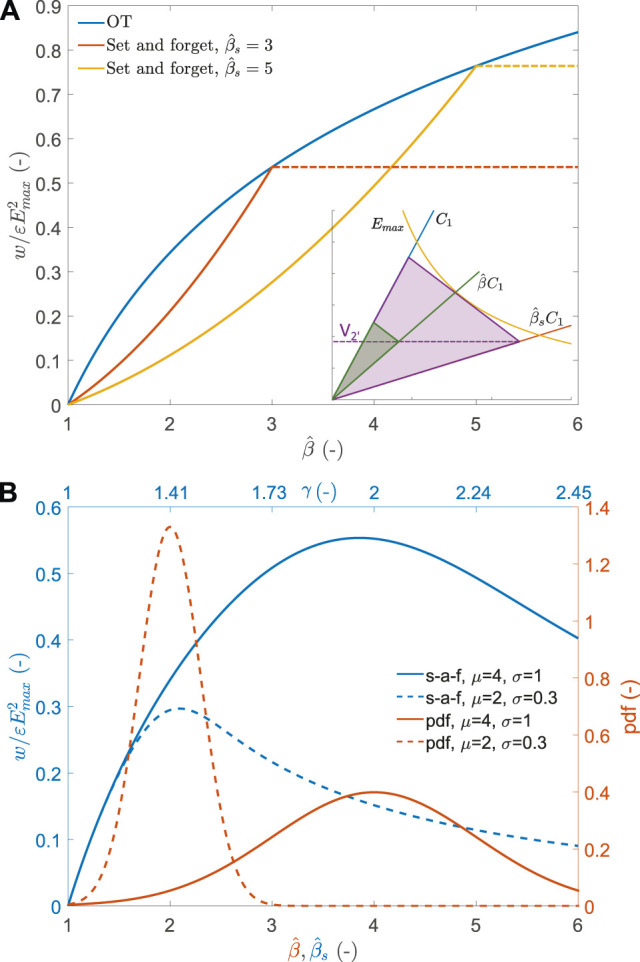
**(A)** normalised energy density harvested as a function of capacitance swing $\hat{\beta }$ using the set-and forget strategy. Two different arbitrary set points are shown: a circuit optimised for ${\hat{\beta }}_{s}=3$ (red) and one for ${\hat{\beta }}_{s}=5$ (yellow). The energy density of the OT cycle is shown in blue as a comparison. inset: representation of the set-and-forget cycle on the Q-V plane. The circuit is optimised for the purple cycle, but a smaller amplitude leads to the green triangle. **(B)** Red: probability density function (pdf) for two normal distributions of capacitance swing. Blue: average energy harvested per cycle for the same distribution of capacitance swing, as a function circuit set point ${\hat{\beta }}_{s}$ .

Figure 6A illustrates the normalised energy density generated per cycle for the set-and-forget approach. The red and yellow curves show the output energy density as a function of the capacitance swing of the device for two arbitrary values of the functioning set point at which the harvesting circuit (value of storage capacitor *C*_*s*_ and priming voltage *V*_2’_) is tuned. The red curve is for a circuit optimised at ${\hat{\beta }}_{s}=3$ , and the yellow curve for ${\hat{\beta }}_{s}=5$ . The energy output obtained for an OT cycle is shown in blue for comparison. The horizontal dashed lines for both set-and-forget curves illustrates the mechanical stop that keeps the harvester in the zone $\hat{\beta }\leq {\hat{\beta }}_{s}$ , thus avoiding exceeding the maximal admissible field in the structure *E*_max_. It illustrates that a mechanical energy input that would cause a capacitance swing $\hat{\beta }>{\hat{\beta }}_{s}$ is effectively limited to ${\hat{\beta }}_{s}$ by the mechanical stop, and the produced energy saturates. The inset of the figure illustrates the set-and-forget cycle: the electronic circuit is set according to the OT cycle equations for a capacitance swing ${\hat{\beta }}_{s}$ , which leads to a harvesting cycle described by the purple triangle. An effective input of amplitude $\hat{\beta }<{\hat{\beta }}_{s}$ leads to the green cycle due to the fixed value of the priming voltage *V*_2’_ and slope of the relaxation phase. This cycle is sub-optimal, as *E*_max_ is never reached. Figure 6A shows that unless the deformation amplitude of the DEG is exactly that for which the circuit has been tuned, the amount of harvested energy is lower than the OT cycle.

We now consider a source of mechanical energy with non-constant amplitude acting on the generator. As an illustration, we use a normal distribution of input capacitance swing $\hat{\beta }$ with a mean value *μ* and a standard deviation *σ*, and look at the energy density generated as a function of the set point ${\hat{\beta }}_{s}$ (Figure 6B). The red curves show two examples of normal distributions with different mean and standard deviation values, and the blue curves show the respective average normalised energy density harvested per cycle as a function of ${\hat{\beta }}_{s}$ . Choosing ${\hat{\beta }}_{s}$ implies setting the priming voltage according to Eq. (3), and choosing the storage capacitor. The top axis of the figure indicates the ratio of the storage capacitor *γ* = *C*_*s*_/*C*_1_. It can be seen that the optimal value of ${\hat{\beta }}_{s}$ is very close to that of the mean value of the distribution (actually, slightly lower, especially for distribution with a larger standard deviation). With the optimal circuit parameters, the normalised harvested energy is 0.30 and 0.55 for the distribution with a mean of 2 and 4, which represents respectively 88 and 81% of what a constant field cycle could harvest for the same input distribution. Although that may appear to be a large fraction of the available energy for a straightforward circuit, these values are only reached if the harvesting circuit is designed at the optimal value of ${\hat{\beta }}_{s}$ , which assume a good knowledge of the input distribution. The harvested energy falls quickly otherwise, especially if ${\hat{\beta }}_{s}$ is chosen too low. Supplementary Figure S3 shows that with a properly tuned circuit, the harvested energy is between 81 and 90% of the maximal harvestable energy of the constant field (CE) cycle.

### Adaptive Harvesting Cycle

Although the previous section has shown that the set-and-forget approach can harvest amounts of energy close to that of the CE cycle, this is only possible for a well-tuned circuit, and therefore requires good knowledge of the statistical distribution of the deformation applied to the harvester. An alternative approach consists in adapting the harvesting circuit to the deformation amplitude. This requires continuous measurement of the deformation of the generator, either using external sensors or a self-sensing scheme (Rizzello et al., 2018). As part of the generated energy needs to be used to monitor the deformation, this approach is more adapted to high-power generators, such as wave energy converters (Moretti et al., 2020a). Out of the two parameters of the harvesting circuit, the value of the storage capacitor *C*_*s*_ is fixed and cannot adapt to the varying deformation amplitude. However, the priming voltage *V*_2’_ can be adjusted on the fly to optimise the circuit, i.e. by making sure that the electric field in the generator reaches *E*_max_ at one point of the cycle. This is done by implementing the strategy described in the “*Non-optimal storage capacitor*” section. Unlike the set-and-forget approach, there is no need to limit the deformation of the generator mechanically, as a large deformation will be compensated by a lower priming voltage so that *E*_max_ is not exceeded.

**FIGURE 7:**
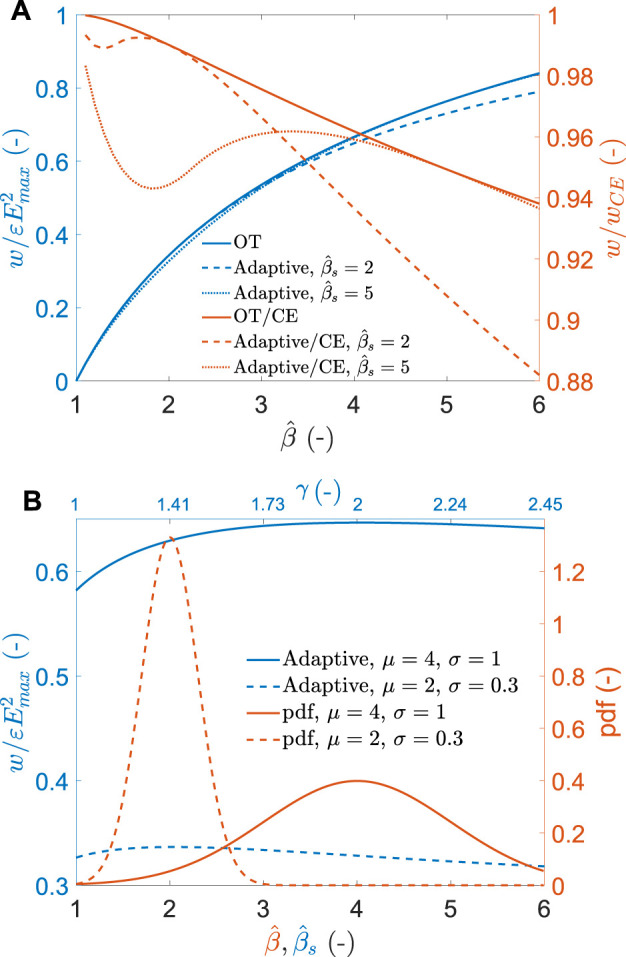
**(A)** normalised energy density harvested as a function of capacitance swing $\hat{\beta }$ using the adaptive strategy (blue). Two different arbitrary set points are shown: a circuit optimised for ${\hat{\beta }}_{s}=2$ (dotted) and one for ${\hat{\beta }}_{s}=5$ (dashed). The energy density of the OT cycle is shown (continuous) as a comparison. Red shows the relative energy produced compared to the CE cycle. **(B)** Red: two normal distributions of capacitance swing. Blue: average energy harvested per cycle for the same distributions of capacitance swing, as a function of the electronic circuit set point ${\hat{\beta }}_{s}$ .

Figure 7A shows in blue the relative amount of energy density that can be harvested using the adaptive approach. Two arbitrary values of ${\hat{\beta }}_{s}$ are chosen (dashed and dotted lines) and compared to the OT cycle (continuous line), and the relative amount of energy with respect to the CE cycle is shown in red for the 3 cases. Over the range of capacitance swings shown on the graph, the adaptive approach allows harvesting an amount of energy that is very close to that of the OT cycle. Even when tuned for a capacitance swing of 2, the adaptive approach enables to harvest 88% of the available energy at a capacitance swing of 6. This means that the adaptive approach remains efficient even when the deformation amplitude of the DEG is not constant. This is shown in Figure 7B, which shows the average normalised energy density harvested per cycle as a function of the circuit set point ${\hat{\beta }}_{s}$ (blue) for a range of deformation amplitude following two normal distributions with different parameters (red). The optimal circuit parameters are obtained when the set point ${\hat{\beta }}_{s}$ is equal to the mean value of the normal distribution. In this situation, the normalised harvested energy is 0.33 and 0.65 for the distributions with a mean of 2 and 4, which represents respectively 97 or 96% of what a CE cycle could harvest for the same input distributions. Furthermore, even if the value of ${\hat{\beta }}_{s}$ does not exactly match the peak of the distribution, the impact on the harvested energy is minimal. This is in stark contrast with the set-and-forget approach (Figure 6B), for which the normalised energy falls quickly if the value of ${\hat{\beta }}_{s}$ does not precisely match the optimal value. Consequently, the adaptive approach leads to a slightly higher energy collection than the set-and-forget approach when the methods are used with optimal parameters. Furthermore, it is much less dependent on the distribution parameters’ knowledge and is thus more versatile.

## Experimental Verification

**FIGURE 8:**
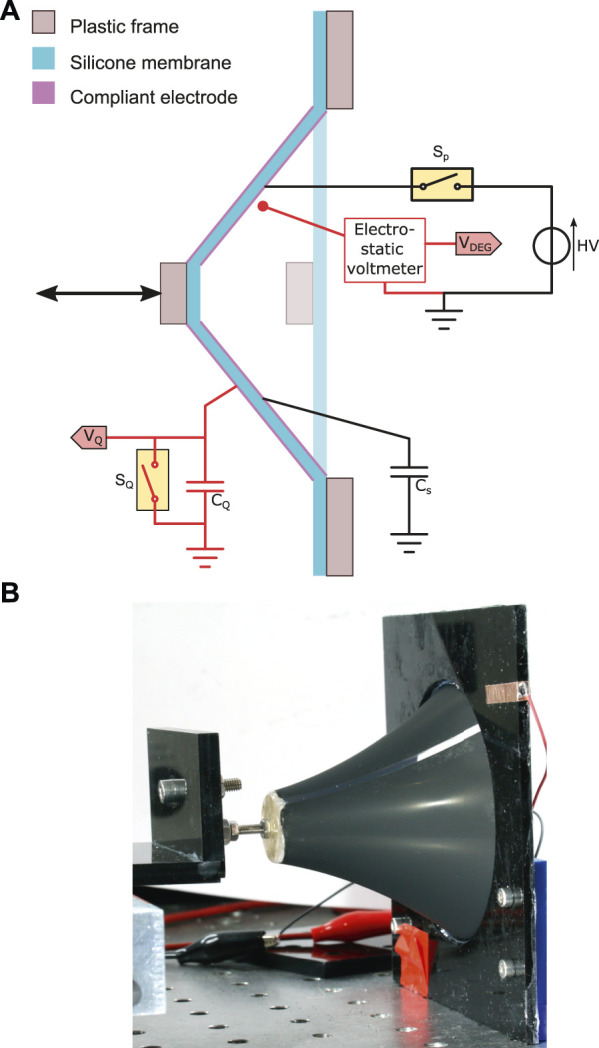
**(A)** schematic representation of the conical DEG under test. The parts in red are components used to characterised the behaviour of the DEG, but which are not required for normal operation. **(B)** picture of the device under test with an out-of-plane displacement of the central hub of 60 mm.

To illustrate the usage of the model, we produced a circular DEG deforming out of plane like a cone (Figure 8). The outer diameter of the membrane was 80 mm with a circular central hub of 20 mm in diameter. The membrane material was a 50 µm sheet of Elastosil 2030 (Wacker Chemie AG) on which compliant electrodes were patterned by spray-coating through a shadow mask. The initial capacitance of the DEG was 2.25 nF, the surface of active material in the undeformed state *S*_1_ was 4.71 ⋅ 10^–3^ m^2^, and the volume of active material was 2.36 ⋅ 10^–7^ m^3^. These values lead to a relative permittivity of the silicone membrane of *ɛ*_*r*_ = 2.7 (the manufacturer’s value provided in the datasheet is 2.8). A servo-tube was connected to the central hub of the DEG and could pull the central hub out of plane. Displacements up to 70 mm were performed leading to achievable values of capacitance swing in the range $1\leq \hat{\beta }\leq 4.5$ . Priming charges were provided by a computer-controlled Peta-pico-Voltron high-voltage power supply (Schlatter et al., 2018) through the priming relay *S*_*p*_. Although a diode would have been sufficient, we used a relay, which has no leakage current, to perform a precise characterisation of the harvesting cycle of the DEG. The storage capacitor *C*_*s*_ enables to generate the triangular harvesting cycle (c.f. “The energy harvesting cycle” section). The range of capacitors that we used for testing, as well as the corresponding value of *γ* = *C*_*s*_/*C*_1_, and the corresponding capacitance swing $\hat{\beta }$ for which the capacitor is optimal is given in Table 3.

**TABLE 3:** Values of storage capacitors available for the experiments, and corresponding values of parameter *γ*, and $\hat{\beta }$ (i.e., the capacitance swing for which this storage capacitor is optimal.).

*C*_*s*_ (nF)	2.2	2.74	3.3	4.7	6.8
*γ* (−)	0.98	1.22	1.47	2.09	3.02
$\hat{\beta }$ (−)	—	1.49	2.15	4.36	9.12

It is necessary to measure the voltage (V) and charge (Q) on the DEG during a cycle to characterise the harvesting cycle, which requires the use of additional electronic components (in red in Figure 8). We used a non-contact electrostatic voltmeter (Trek P0865) to measure the voltage, which provided voltage reading without charge leakage. To measure the charge on the DEG, we connected the DEG in series with a large capacitor *C*_*Q*_ of 16.8 µF (i.e. 7500 times larger than the capacitance of the DEG). The voltage *V*_*Q*_ on this sensing capacitor is proportional to the charge on the DEG *Q*_*DEG*_ according to the equation *Q*_*DEG*_ = *C*_*Q*_ *V*_*Q*_. The voltage *V*_*Q*_ was buffered through an operational amplifier with a high input impedance to reduce charge leakage. Still, as leakage cannot be prevented entirely, a relay *S*_*Q*_ was connected in parallel with *C*_*Q*_ and enabled to discharge the integrator before each cycle. An Analog Discovery 2 was used to read the electrostatic voltmeter output and the voltage *V*_*Q*_, and to control the two relays. A LabVIEW programme controlled the whole setup, including relay state, priming voltage value, servo-tube position, and data acquisition. For all test cycles, the speed of the servo tube was adapted so that the stretching phase (and the relaxing phase) took 1 s. A LCR meter was used to measure the capacitance of the DEG as a function of the servo-tube displacement to establish a relation between the capacitance swing and physical displacement of the DEG. Five cycles were measured for displacements up to 70 mm and a theoretical model fitted on the experiment (see Supplementary Material). This model was later used in the experiments to prescribe the servo-tube displacement required to achieve a target capacitance swing $\hat{\beta }$ .

To calculate the measured energy density *w*_*meas*_ collected during each cycle, we used the charge and voltage measured at the end of priming ( $Q_{2}^{\prime }$ , $V_{2}^{\prime }$ ), and the charge and voltage measured at the end of relaxation ( $Q_{1}^{\prime }$ , $V_{1}^{\prime }$ ) (c.f. Figure 3 or Supplementary Figure S1 for location of points 1′ and 2′). The harvested energy density (i.e. the area included within the triangular harvesting cycle is therefore given by:

$$w_{meas}=\frac{\left(V_{2}^{\prime }+V_{1}^{\prime }\right)\left(Q_{2}^{\prime }-Q_{1}^{\prime }\right)}{2\Omega }+\frac{Q_{1}^{\prime }V_{1}^{\prime }}{2\Omega }-\frac{Q_{2}^{\prime }V_{2}^{\prime }}{2\Omega }$$ (8)

The first term is the energy density transferred to the storage capacitor during the relaxation phase. The second term is the energy density extracted from the DEG during the discharge phase. The third term is the priming energy density of the DEG capacitance. The priming energy of the storage capacitor does not appear, as it is also collected during the discharge phase and only acts as an offset.

## Results

**FIGURE 9:**
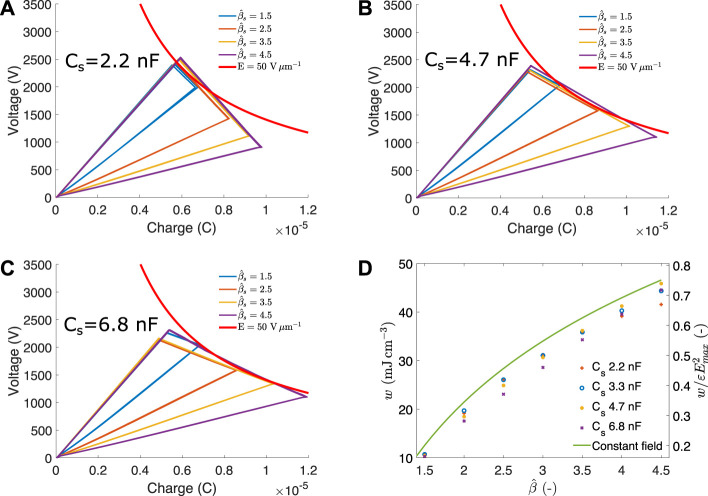
Harvesting cycles for capacitance swings between 1.5 and 4.5 and different values of storage capacitor: **(A)** 2.2 nF, **(B)** 4.7 nF, **(C)** 6.8 nF. **(D)** average energy density and normalised energy density as a function of capacitance swing and for different values of storage capacitor. The maximal theoretical harvestable energy density as provided by the constant field cycle is also indicated for reference.

Harvesting cycles were measured for a range of capacitance swings $1.5\leq \hat{\beta }\leq 4.5$ , and for four different storage capacitor values (Figure 9). The maximal field was set to *E*_max_ = 50 V µm^−1^ to stay clear from the *E*_max_ = 80 V µm^−1^ breakdown field given by the manufacturer. Priming voltage values were calculated using the expressions from table 2 to construct the best triangular harvesting cycle for the given values of $\hat{\beta }$ and *γ*, and not exceeding a field of 50V µm^−1^. Five harvesting cycles were performed and are displayed on the graphs for each value of $\hat{\beta }$ . The five cycles overlap almost perfectly, showing a very high reproducibility. The red line represents the maximal electric field, with points on the right having a higher electric field value.

The smallest value of *C*_*s*_ = 2.2 nF (Figure 9A) leads to a value of *γ* < 1 (c.f. Table 3). Consequently, the triangular cycles for this value of *C*_*s*_ are not tangent to the maximal field line but meet it at the end of the relaxation phase (c.f. Supplementary Figure S1, case *C*_*s*1_). A value of *C*_*s*_ = 4.7 nF (Figure 9B) is optimal for a capacitance swing of 4.36, close to the cycle with $\hat{\beta }=4.5$ . This cycle is tangent to the maximal field line at the middle of the relaxation phase and leads to a very close cycle to the ideal constant field cycle. As *γ* = 2.09, the cycle to $\hat{\beta }<2.09$ (i.e. the cycle to 1.5) is not tangent to the maximal electric field line but touches it at the end of the priming phase (c.f. Supplementary Figure S1, case *C*_*s*3_). Finally, the largest storage capacitor *C*_*s*_ = 6.8 nF is too large for the range of capacitance swing tested here. This leads to cycles with a capacitance swing of 1.5 and 2.5 to touch the maximal field line at the end of the priming voltage. Cycles with a capacitance swing of 3.5 and 4.5 become tangent with the maximum field line at the beginning of their relaxation. The results of Figure 9 show that the experimental DEG behaves as expected and that the model enables to design a triangular harvesting cycle that approaches *E*_max_ without exceeding it. The complete set of cycles for all tested values of $\hat{\beta }$ and *C*_*s*_ is shown in Supplementary Figure S5.

Figure 9D shows the energy density (left axis) and normalised energy density (right axis) generated for the seven capacitance swing values tested and the four storage capacitor values. Each data point is the average of the five harvesting cycles performed for each combination. The green line represents the maximum harvestable energy density of a constant field cycle. If the priming voltage is judiciously chosen with respect to the values of *C*_*s*_ and $\hat{\beta }$ , the triangular cycle enables to harvest a large portion of the harvestable energy. *C*_*s*_ = 3.3 nF is the optimal storage capacitor value for $\hat{\beta }=2.15$ , and it experimentally leads to the highest harvested energy value for $\hat{\beta }=2$ , compared to the other capacitor values. Similarly, *C*_*s*_ = 4.7 nF is the optimal storage capacitor value for $\hat{\beta }=4.36$ , and it experimentally leads to the highest harvested energy value for $\hat{\beta }=4.5$ , compared to the other capacitor values. Supplementary Figure S7 shows the energy density of each measurement point relative to that of the constant field cycle, which represents the highest quantity of electrical energy that can be collected. Selecting the storage capacitor closest to the optimal value, the relative quantity of energy collected during this series of tests represents 86–98% of the energy that can be collected.

To evaluate the energy harvesting performance of the DEG for varying deformation amplitudes, we measured the harvested energy density for series of 200 cycles with a capacitance swing following a normal distribution. As the DEG had been successfully tested for values of $\hat{\beta }=4.5$ , we centred the distribution on $\hat{\beta }=2.75$ , with a standard deviation of 0.8. This ensured that 97% of the cycles were within the range of capacitance swings $1\leq \hat{\beta }\leq 4.5$ . Each test was performed with a different set of random values. During a test, capacitance swings were performed in the order they were generated (i.e. not sorted in increasing capacitance swing values). Supplementary Figure S5 shows a representative set of capacitance swings used for the characterisation.

**FIGURE 10:**
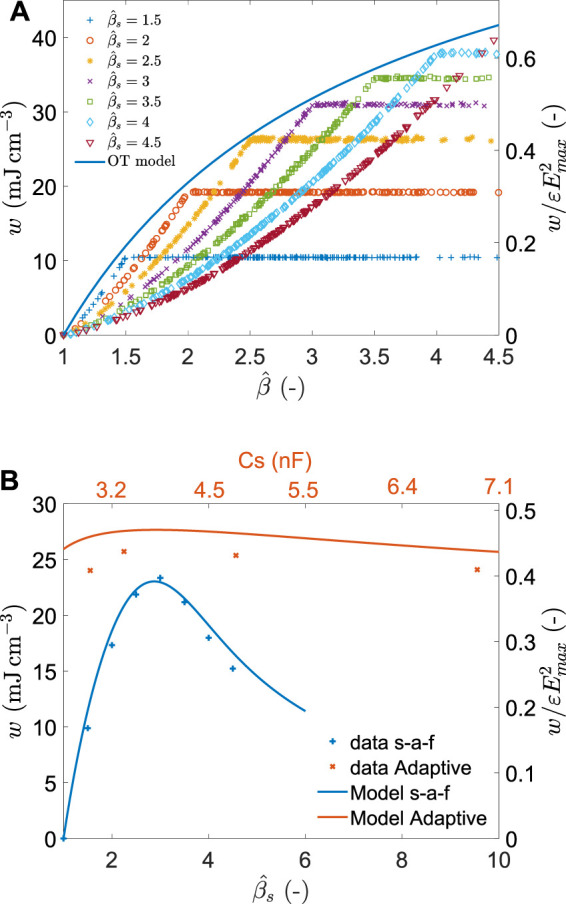
**(A)** Energy density (left axis) and normalised energy density (right axis) obtained using the set-and-forget scheme for different values of $\hat{\beta_{s}}$ , and 200 cycles for each value, randomly chosen with a normal distribution with *μ* = 2.75, and *σ* = 0.8. **(B)** Average energy density (left axis) and normalised energy density (right axis) per cycle calculated on 200 cycles following a normal distribution with *μ* = 2.75, and *σ* = 0.8. Blue is using the set-and-forget approach, and red is using the adaptive harvesting cycle approach. The upper *x* axis indicate the value of *C*_*s*_ corresponding to $\hat{\beta_{s}}$ for the Adaptive scheme.

We started by considering the set-and-forget approach with functioning set-points in the range $1.5\leq \hat{\beta_{s}}\leq 4.5$ with steps of 0.5. The storage capacitance was set to 3.3 nF for all set point values, thus fixing *γ* = 3.3/2.25 = 1.47. The priming voltage was then defined using Table 2. One set of 200 cycles was performed for each of the functioning set points. Each capacitance swing value in the testing set represents the capacitance change imposed to the DEG for a given mechanical energy input. However, as detailed in section 4.1, an input of mechanical energy that causes a capacitance swing higher than $\hat{\beta_{s}}$ would cause the electric field in the device to exceed the limit *E*_max_, and consequently, the set-and-forget cycle must include mechanical stops to prevent the DEG to reach a capacitance swing exceeding $\hat{\beta_{s}}$ . Figure 10A) shows the measured energy density for the 200 cycles of each value of $\hat{\beta_{s}}$ . The energy density predicted for an OT cycle is shown as a continuous curve. As detailed in section 4.1, the energy density of the set-and-forget scheme is expected to reach the OT cycle for $\hat{\beta }=\hat{\beta_{s}}$ . For larger values $\hat{\beta }$ , the mechanical stop prevents the effective capacitance swing to exceed $\hat{\beta }$ and the energy output saturates. We observe that the experimental data closely matches the predicted values. The average energy density per cycle for each value of $\hat{\beta_{s}}$ is shown on Figure 10B) together with the predicted value from the model. There is an excellent agreement between the model and the measured data. For a $\hat{\beta_{s}}$ value of 3 (i.e. the closest value to the mean of the normal distribution), the average energy density per cycle reaches 23.3J cm^−3^.

We also compared the average harvested energy when an adaptive cycle approach is used instead of the set-and-forget. This was done by selecting a functioning point $\hat{\beta_{s}}$ through the choice of the storage capacitance *C*_*s*_ (c.f. section 4.2), while adapting the priming voltage $V_{2}^{\prime }$ to the amplitude of each cycle using the expression of Table 2. This requires some sensing mechanism, either self sensing of the DEG capacitance, or measurement of the mechanical deformation and application of a model linking deformation to capacitance swing. Here, we used the encoder of the servo-tube as a measurement of the displacement amplitude, and calculated the capacitance using our model fitted on experimental data (see Supplementary Figure S4). Similarly to the set-and-forget tests, we used sets of 200 harvesting cycles with capacitance swings randomly distributed according to a normal distribution with *μ* = 2.75, and *σ* = 0.8. Four values of storage capacitor were used: 2.74, 3.3, 4.7, and 6.8 nF, corresponding to capacitance swing set points as indicated in Table 3. The four sets of 200 values are shown in Supplementary Figure S8, and the average amount of energy density collected by cycle is shown in Figure 10B). The highest average amount of energy density is observed for $\hat{\beta_{s}}=2.15$ (*C*_*s*_ = 3.3 nF), the closest value from the average of the distribution. The collected energy density for this configuration is 25.7 J cm^−3^, i.e. slightly higher than the peak value measured for the set-and-forget approach. However, using storage capacitors that deviate from the optimal value leads to little change in collected energy density, as predicted by the model. The experimental values are 7% lower than what the model predicts. The energy density harvested using these two schemes is indicated in Table 4 and compared to the maximal possible harvestable energy provided by the CE field.

**TABLE 4:** Energy density harvested at a field of 50 V µm^−1^ for an input distribution of capacitance swing values following a normal distribution with *μ* = 2.75, and *σ* = 0.8. The constant field (CE) value is a theoretical value, and the set-and-forget (s-a-f) and adaptive values are the experimental values obtained as the average of 200 cycles. The second line shows the relative energy density wrt the CE benchmark.

Harvesting scheme	CE	s-a-f	Adaptive
*w* (mJcm^−3^)	29.9	23.3	25.7
*w*/*w*_*CE*_ (−)	—	0.78	0.86

## Discussion

The DEG is a generator technology that scales well and can therefore be integrated at different size scales. For example, concepts of large-scale ( > 1 kW) DEG-based wave energy converters have been proposed, with the concept demonstrated on smaller-size prototypes, such as oscillating bodies (Kornbluh et al., 2011), oscillating water columns (Moretti et al., 2018), or attenuators (Jean et al., 2012). However, smaller wave energy converters producing a fraction of a watt or a few watts—enough to power a few sensors and data telemetry—would be interesting to deploy a sensor network of buoys to monitor water quality, fishing activity, biodiversity, etc. Outside of the water, tree branch motion can be used as a power source for a sensor network monitoring forest health (Anderson et al., 2011), or human body motion, such as heel strike (Kornbluh et al., 2011; Savage, 2012) or knee bending (Lagomarsini et al., 2019) to power physiological sensors. For these smaller-scale applications, cycles that do not require capacitance monitoring and active voltage control during the relaxation phase are advantageous. The OT triangle cycle is not the only harvesting cycle meeting these conditions, and cycles such as constant charge (CC) (c.f. section 2), constant voltage (CV) (Graf et al., 2010), and rectangular (constant charge constant voltage, CCCV) (Kaltseis et al., 2014; McKay et al., 2015; Huang et al., 2013) are also possible. Normalised energy density values for an arbitrary capacitance swing of $\hat{\beta }=2.5$ for the four cycles mentioned previously are listed in Table 5, and compared to the constant field cycle. The OT triangle exhibits a much better performance than the other cycles without being more difficult to implement. In previous work, we tested a miniaturised stacked DEA using a CCCV cycle (McKay et al., 2015). At a working field of *E*_max_ = 50V m^−1^ and for a capacitance swing of 2, the stacked DEG produced and energy of 0.5 mJ/cycle. The generator had a volume of 0.32 cm^−3^, leading to an energy density per cycle of 1.56 mJcm^−3^. In comparison, the DEG tested in this contribution produced an energy density of 19.7 mJcm^−3^ for the same electric field and capacitance swing, a factor 12.6 increase.

**TABLE 5:** Representative values of normalised energy density and relative energy density (wrt constant field), for different harvesting cycles. The constant field (CE) cycle is given as reference value. The values are calculated using the formulas from Moretti et al. (2020a).

Cycle	CE	CC	CV	OT	CCCV
$w/\varepsilon E_{max}^{2}$ at $\hat{\beta }=2.5$ (−)	0.458	0.300	0.300	0.450	0.135
*w*/*w*_*CE*_ at $\hat{\beta }=2.5$ (−)	—	0.655	0.655	0.982	0.295

Passive harvesting cycles are optimised for a precise deformation (i.e. capacitance swing) of the DEG. However, real-life applications of DEGs usually involve deformation amplitudes that can change with time. We have considered the case of a normal input distribution of capacitance swing values and experimentally validated the model with tests performed at an electric field *E*_max_ = 50 V m^−1^ and for distribution parameters of *μ* = 2.75, and *σ* = 0.8. In these conditions, the completely passive set-and-forget approach enables to harvest 78% of what would be collected with the CE cycle (c.f. Table 4). This requires knowing the parameters of the distribution so that the cycle can be tuned accordingly. This is a realistic assumption; for example, wave amplitudes throughout the year can be measured at the location where a wave energy converter will be installed and the amplitude distribution established. In the same conditions, the adaptive cycle enables to harvest slightly more energy (86% of the CE cycle). However, it requires active monitoring of the deformation to adapt the priming voltage, which defeats the idea of a completely passive harvesting cycle. Still, compared to the CE cycle that requires monitoring the capacitance of the DEG during the whole relaxation phase and controlling the voltage to ensure that the electric field remains constant, the adaptive cycle only requires a single measurement of the peak deformation, which can potentially be done at a much lower energy cost.

Other types of distributions will affect the results, but the set of equations developed here can easily be applied to predict the performance of a DEG for any kind of input distribution. It is, for example, expected that a uniform distribution would be detrimental to the set-and-forget approach, as a larger fraction of the cycles would be located further apart from the capacitance swing for which the circuit is tuned. Still, the performance of the set-and-forget approach can be compared to what would be collected by the adaptive approach, taking into account the energy required for the deformation monitoring system to decide which configuration is better suited.

The cycles performed at $\hat{\beta }=4.5$ with a maximal electric field of 50V µm^−1^ collected 46 mJcm^−3^ (Figure 9D). This represents 98% of the harvestable energy. It is important to point out that the notion of *fraction of harvestable energy* represents the quantity of electrical energy collected with respect to that collected with the ideal constant field cycle. It does not represent the conversion factor of the harvester in terms of collected electrical energy versus mechanical energy input. The DEG efficiency depends on its mechanical design, impedance matching and other parameters. Here, we only consider the energy collected for the chosen values of electric field and deformation, without considering the mechanical energy required to provide this deformation. With a similar generator (same membrane material, but using bi-axial extension), Moretti et al. measured conversion factors up to 30% (Moretti et al., 2017).

One of the main drawbacks of the approach presented here is that it requires a substantial priming energy for each cycle. As shown on Figure 5A, the net energy gain per cycle can be smaller than the priming energy, especially for large values of *C*_*s*_ and low values of $\hat{\beta }$ . The requirement to reach *E*_max_ at one point of the cycle to maximise the energy gain, coupled with the typical thickness of DEG membranes (50–100 µm) means that this priming energy must be delivered at high voltage (the experiments used priming voltages between 1000 and 2000 V). This can make the design of a priming source challenging for small-scale applications. If the priming source is unable to deliver enough energy to prime the DEG to its optimal voltage value, the harvesting cycle will only reach a fraction of *E*_max_ and lead to a drastic reduction of the collected energy, due to its quadratic dependence with the electric field [c.f. Eq. (6)]. In these situations, the self-priming configuration introduced by McKay et al. can be an interesting alternative (McKay et al., 2010b,a). It uses a small initial priming energy which is exchanged back and forth between the DEG and a storage capacitor (or between the two sides of a DEG in its integrated implementation), slowly building up voltage until the maximal field is reached. Finding the optimal implementation (OT or integrated self-priming circuit) that leads to the largest amount of harvested energy depends on the capacitance swing and the energy available from the priming source. This is an interesting problem and will be the subject of a future contribution.

## Conclusion

We have established a model that maximises the energy harvested by a simple circuit consisting of a DEG and a storage capacitor. The model gives the values of the required storage capacitor and the priming voltage, which maximises the collected energy while keeping the electric field in the device lower than a set value. For a range of capacitance swing values <6 , the predicted energy that can be collected using this harvesting scheme is larger than 94% of the harvestable energy. This *optimal triangle* scheme requires the use of a storage capacitor that matches the deformation amplitude of the DEG, which is impractical as the deformation amplitude of a DEG can change with time. However, we have demonstrated that a close-to-optimal harvesting cycle can be performed provided that the value of the storage capacitor is known. We have developed a set of equations to calculate the priming voltage and generated energy using the storage capacitor as an additional parameter.

The model has been validated by experiments on a conical DEG and can be used to optimise the energy collected by a DEG in the case of non-constant deformation amplitudes. At a conservative electric field value of 50V µm^−1^, an energy density up to 46 mJcm^−3^ was generated. This represents 98% of the energy that would be collected using a constant field cycle, which would require complex and potentially energy-expensive control of the DEG voltage during relaxation. The economy of the OT scheme makes it near ideal for small, portable, wearable and natural stochastic energy harvesting operations.

## Data Availability

The raw data supporting the conclusion of this article will be made available by the authors, without undue reservation.

## References

[B1] Albuquerque F. B. Shea H. (2020). Influence of Humidity, Temperature and Prestretch on the Dielectric Breakdown Strength of Silicone Elastomer Membranes for DEAs. Smart Mater. Struct. 29, 105024. 10.1088/1361-665X/aba5e3

[B2] Anderson I. A. Gisby T. A. McKay T. G. O’Brien B. M. Calius E. P. (2012). Multi-functional Dielectric Elastomer Artificial Muscles for Soft and Smart Machines. J. Appl. Phys. 112, 041101. 10.1063/1.4740023

[B3] Anderson I. A. Ieropoulos I. A. McKay T. O'Brien B. Melhuish C. (2011). Power for Robotic Artificial Muscles. Ieee/asme Trans. Mechatron. 16, 107–111. 10.1109/tmech.2010.2090894

[B4] Bruch D. Nalbach S. Rizzello G. Motzki P. Seelecke S. (2020). “Multifunctional Fatigue Testing Setup for In-Plane Operating DEAs,”. Nondestructive Characterization and Monitoring of Advanced Materials, Aerospace, Civil Infrastructure, and Transportation IX. Editors Shull P. J. Yu T.-Y. Gyekenyesi A. L. Wu H. F. (Bellingham, WA: SPIE), 11380, 113800S. 10.1117/12.2558609

[B5] de Saint-Aubin C. A. Rosset S. Schlatter S. Shea H. (2018). High-cycle Electromechanical Aging of Dielectric Elastomer Actuators with Carbon-Based Electrodes. Smart Mater. Struct. 27, 074002. 10.1088/1361-665x/aa9f45

[B6] Eitzen L. Graf C. Maas J. (2011). Cascaded Bidirectional Flyback Converter Driving DEAP Transducers. IECON 2011 - 37th Annual Conference of the IEEE Industrial Electronics Society. IEEE, 1226–1231. 10.1109/iecon.2011.6119484

[B7] Fasolt B. Welsch F. Jank M. Seelecke S. (2019). Effect of Actuation Parameters and Environment on the Breakdown Voltage of Silicone Dielectric Elastomer Films. Smart Mater. Struct. 28, 094002. 10.1088/1361-665x/ab2f34

[B8] Graf C. Eitzen L. Maas J. (2011). Multilevel High Voltage Converter Driving Dielectric Elastomer Generators. In Proceedings of the 2011 14th European Conference on Power Electronics and Applications. 1–10.

[B9] Graf C. Maas J. Schapeler D. (2010). Energy Harvesting Cycles Based on Electro Active Polymers. In Proceedings of SPIE - The International Society for Optical Engineering (SPIE), 7642, 764217. 10.1117/12.853597

[B10] Huang J. Shian S. Suo Z. Clarke D. R. (2013). Maximizing the Energy Density of Dielectric Elastomer Generators Using Equi-Biaxial Loading. Adv. Funct. Mater. 23, 5056–5061. 10.1002/adfm.201300402

[B11] Jean P. Wattez A. Ardoise G. Melis C. Van Kessel R. Fourmon A. (2012). Standing Wave Tube Electro Active Polymer Wave Energy Converter. Proc. SPIE - Int. Soc. Opt. Eng. 8340, 83400C. 10.1117/12.934222

[B12] Jean-Mistral C. Basrour S. Chaillout J.-J. (2008). “Dielectric Polymer: Scavenging Energy from Human Motion,”. Electroactive Polymer Actuators and Devices (EAPAD) 2008. Editor Bar-Cohen Y. (Bellingham, WA: SPIE), 6927, 692716. 10.1117/12.776879

[B13] Kaltseis R. Keplinger C. Koh S. J. A. Baumgartner R. Goh Y. F. Ng W. H. (2014). Natural Rubber for Sustainable High-Power Electrical Energy Generation. RSC Adv. 4, 27905–27913. 10.1039/c4ra03090g

[B14] Koh S. Keplinger C. Li T. Bauer S. Suo Z. (2011). Dielectric Elastomer Generators: How Much Energy Can Be Converted? Mechatronics, IEEE/ASME Trans. 16, 33–41. 10.1109/TMECH.2010.2089635

[B15] Kornbluh R. D. Pelrine R. Prahlad H. Wong-Foy A. McCoy B. Kim S. (2011). From Boots to Buoys: Promises and Challenges of Dielectric Elastomer Energy Harvesting. Proc. SPIE - Int. Soc. Opt. Eng. 7976, 797605. 10.1117/12.882367

[B16] Lagomarsini C. Jean-Mistral C. Monfray S. Sylvestre A. (2019). Optimization of an Electret-Based Soft Hybrid Generator for Human Body Applications. Smart Mater. Structures 28, 104003. 10.1088/1361-665x/ab3906

[B17] Lo H. C. (2015). Converters for Milliwatt Dielectric Elastomer Generators. Ph.D. thesis. Auckland, New Zealand: The University of Auckland.

[B18] McKay T. G. Rosset S. Anderson I. A. Shea H. (2015). Dielectric Elastomer Generators that Stack up. Smart Mater. Structures 24, 015014. 10.1088/0964-1726/24/1/015014

[B19] McKay T. O’Brien B. Calius E. Anderson I. (2010a). An Integrated, Self-Priming Dielectric Elastomer Generator. Appl. Phys. Lett. 97, 062911. 10.1063/1.3478468

[B20] McKay T. O’Brien B. Calius E. Anderson I. (2010b). Self-priming Dielectric Elastomer Generators. Smart Mater. Structures 19, 055025. 10.1088/0964-1726/19/5/055025

[B21] Moretti G. Herran M. S. Forehand D. Alves M. Jeffrey H. Vertechy R. (2020a). Advances in the Development of Dielectric Elastomer Generators for Wave Energy Conversion. Renew. Sustainable Energ. Rev. 117, 109430. 10.1016/j.rser.2019.109430

[B22] Moretti G. Papini G. P. R. Righi M. Forehand D. Ingram D. Vertechy R. (2018). Resonant Wave Energy Harvester Based on Dielectric Elastomer Generator. Smart Mater. Structures 27, 035015. 10.1088/1361-665x/aaab1e

[B23] Moretti G. Righi M. Vertechy R. Fontana M. (2017). Fabrication and Test of an Inflated Circular Diaphragm Dielectric Elastomer Generator Based on PDMS Rubber Composite. Polymers 9, 283. 10.3390/polym9070283 PMC6432430 30970961

[B24] Moretti G. Rosset S. Vertechy R. Anderson I. Fontana M. (2020b). A Review of Dielectric Elastomer Generator Systems. Adv. Intell. Syst. 2, 2000125. 10.1002/aisy.202000125

[B25] Pelrine R. Kornbluh R. Pei Q. Joseph J. (2000). High-speed Electrically Actuated Elastomers with Strain Greater Than 100%. Science 287, 836–839. 10.1126/science.287.5454.836 10657293

[B26] Rizzello G. Seelecke S. Homer M. Rossiter J. de Oliveira Zanini P. R. (2018). “Self-sensing for Robust Automatic Charge Management of Dielectric Elastomer Generators,”. Proceedings of SPIE - the International Society for Optical Engineering. Editor Bar-Cohen Y. (Bellingham, WA: SPIE), 10594, 105941J. 10.1117/12.2295355

[B27] Rosset S. de Saint-Aubin C. Poulin A. Shea H. R. (2017). Assessing the Degradation of Compliant Electrodes for Soft Actuators. Rev. Scientific Instr. 88, 105002. 10.1063/1.4989464 29092503

[B28] Rosset S. Shea H. R. (2016). Small, Fast, and Tough: Shrinking Down Integrated Elastomer Transducers. Appl. Phys. Rev. 3, 031105. 10.1063/1.4963164

[B29] Savage N. (2012). Squishy Power Generators. IEEE Spectr.

[B30] Schlatter S. Illenberger P. Rosset S. (2018). Peta-pico-voltron: An Open-Source High Voltage Power Supply. HardwareX 4, e00039. 10.1016/j.ohx.2018.e00039

[B31] Shian S. Huang J. Zhu S. Clarke D. R. (2014). Optimizing the Electrical Energy Conversion Cycle of Dielectric Elastomer Generators. Adv. Mater. 26, 6617–6621. 10.1002/adma.201402291 25113278

[B32] Todorcevic T. Bauer P. Ferreira J. A. van Kessel R. (2013). Bidirectional Modular Multilevel DC-DC Converter Control and Efficiency Improvements through Separate Module Control Method. In 2013 IEEE Energy Conversion Congress and Exposition. IEEE, 2038–2043. 10.1109/ecce.2013.6646957

[B33] Vu-Cong T. Jean-Mistral C. Sylvestre A. (2013). Electrets Substituting External Bias Voltage in Dielectric Elastomer Generators: Application to Human Motion. Smart Mater. Structures 22, 025012. 10.1088/0964-1726/22/2/025012

