# Supplementary material for: Squeezing More Juice out of Dielectric Elastomer Generators
Source: Front Robot AI. 2022 Feb 11;9:825148. doi: 10.3389/frobt.2022.825148 (PMC8873102; doi:10.3389/frobt.2022.825148)
Supplement: Supplementary file 1 [file Presentation1.PDF]

Supplementary Material – Squeezing more juice out of dielectric elastomer generators

1 GENERAL RELATIONSHIPS

The capacitance swing β is defined as the ratio of the capacitance at any time $C(t)$ with respect to the minimal capacitance C_1 (Note that we use index 1, and not 0 for the minimal capacitance, to take into account that some prestretch might be present in the DEG in the minimal capacitance position, depending on the configuration.):

$$\beta(t) = C(t)/C_1, \tag{S1}$$

with $1 \leq \beta(t) \leq \hat{\beta}$. The capacitance can be expressed as a function of the volume of the dielectric and the surface of the capacitor:

$$C(t) = \frac{\varepsilon S(t)^2}{\Omega} = \frac{\varepsilon S_1^2 \beta(t)}{\Omega}, \tag{S2}$$

with ε the permittivity of the material, S the surface of the deformable capacitor at arbitrary time t, and S_1 the minimal surface of the capacitor at the beginning of the cycle, and Ω the volume of the elastomer, which remains constant during deformation. From equation S2, the surface of the DEG can be expressed as a function of the capacitance swing as: $S(t) = \sqrt{\beta(t)}S_1$, which in turns enables to express the charge Q on the DEG as a function of the electric field E and the capacitance swing β:

$$Q = \varepsilon S E = \varepsilon \sqrt{\beta(t)} S_1 E \tag{S3}$$

and consequently, the voltage on the DEG is defined as:

$$V = \frac{Q}{C} = \frac{\Omega \varepsilon \sqrt{\beta(t)} S_1 E}{\varepsilon S_1^2 \beta(t)} = \frac{\Omega E}{S_1 \sqrt{\beta(t)}} \tag{S4}$$

The cycles considered in this contribution are designed to never exceed a maximal electric field E_{max}. From Eq. S3, we can establish the relation between the capacitance swing and the charge, which describes the deformation of the DEG β^* at which a charge Q^* leads to the field E_{max} in the DEG:

$$\beta^* = \frac{Q^{*2}}{\varepsilon^2 S_1^2 E_{max}^2}. \tag{S5}$$

Using the definition of the capacitance swing (Eq. S1), this leads to:

$$\frac{Q^{*2}}{\varepsilon^2 S_1^2 E_{max}^2} = \frac{C^*}{C_1} = \frac{Q^*}{V^* \cdot C_1} = \frac{Q^* \Omega}{V^* \varepsilon S_1^2}, \tag{S6}$$

which finally enables to write the relation between charge and voltage at which the field in the DEG is equal to E_{max}:

$$Q^* = \frac{\Omega \varepsilon E_{max}^2}{V^*}. \tag{S7}$$

In the Q-V plane, this is the equation of a line separating the region with $E < E_{max}$ on the left, from the region with $E > E_{max}$ on the right. The harvesting cycles must be designed to stay on the left side of this line at any time to guarantee that the electric field remains below the threshold.

2 OPTIMAL TRIANGLE CYCLE

This section presents the equations required to calculate the parameters of the optimal triangle harvesting cycle.

During the relaxation phase of the DEG (segment (C)-(D), c.f. figure 2 of the article), the DEG capacitor C is in parallel with the storage capacitor C_s, and the two are isolated from other components. Therefore, neglecting any leakage, the charge in the system remains constant at a value Q_{tot}. The charge Q stored in the DEG during the relaxation phase is therefore given by:

$$Q = Q_{tot} - C_s V, \tag{S8}$$

thus leading to $dV/dQ = -1/C_s$: the slope of the segment (C)-(D) is inversely proportional to the value of the storage capacitor.

To find the parameters of the optimal triangle cycle, we first establish the relations that describe the charge and voltage of the DEG at points 1 and 2 (c.f. Fig. 3 of the article). We use Eq. S3 and S4, taking into account that $\beta = 1$ at point 1, and $\beta = \hat{\beta}$ at point 2, and that the electric field reaches its maximal value at both points.

	Q	V
Point 1	$Q_1 = \varepsilon S_1 E_{max}$	$V_1 = \dfrac{\Omega E_{max}}{S_1}$
Point 2	$Q_2 = \varepsilon S_1 E_{max}\sqrt{\hat{\beta}}$	$V_2 = \dfrac{\Omega E_{max}}{\sqrt{\hat{\beta}} S_1}$

Table S1. Expression of the charge and voltage of the DEG capacitor at point 1 (relaxed capacitor reaches maximal field) and at point 2 (capacitor at maximal deformation reaches maximal field).

The slope of segment (C)-(D) is defined as:

$$\frac{V_2 - V_1}{Q_2 - Q_1} = -\frac{\frac{\Omega E_{max}}{S_1}\left(1 - \frac{1}{\sqrt{\hat{\beta}}}\right)}{\varepsilon S_1 E_{max}\left(\sqrt{\hat{\beta}} - 1\right)} = -\frac{\Omega}{\varepsilon S_1^2}\frac{1}{\sqrt{\hat{\beta}}} = -\frac{1}{C_1\sqrt{\hat{\beta}}}. \tag{S9}$$

As the slope of the segment (C)-(D) is related to the value of the storage capacitor C_s, it follows that to obtain the desired slope, the value of the storage capacitor must be:

$$C_s = C_1\sqrt{\hat{\beta}}. \tag{S10}$$

To obtain the optimal triangle cycle, one shifts the line (C)-(D) so that is becomes tangent with the maximal electric field line (Eq.S7). First, we calculate the slope of the maximal field line:

$$\frac{dV}{dQ} = -\frac{\Omega \varepsilon E_{max}^2}{Q^2}, \tag{S11}$$

and we then find the point Q_{tg} and V_{tg} at which the slope of the maximal field line is equal to that of the segment between points 1 and 2:

$$-\frac{\Omega\varepsilon E_{max}^2}{Q_{tg}^2} = -\frac{\Omega}{\varepsilon S_1^2}\frac{1}{\sqrt{\hat{\beta}}} \Rightarrow Q_{tg} = \varepsilon E_{max} S_1 \sqrt[4]{\hat{\beta}} \tag{S12}$$

$$V_{tg} = \frac{\Omega\varepsilon E_{max}^2}{Q_{tg}} = \frac{\Omega E_{max}}{S_1 \sqrt[4]{\hat{\beta}}} \tag{S13}$$

The relaxation segment (C)-(D) of the optimal triangle has a slope defined by Eq. S9, and must go through the point Q_{tg} and V_{tg}. We can therefore establish the equation of the line that defines the segment (C)-(D):

$$V_{tg} = -\frac{\Omega}{\varepsilon S_1^2}\frac{1}{\sqrt{\hat{\beta}}} Q_{tg} + \alpha \tag{S14}$$

$$\frac{\Omega E_{max}}{S_1 \sqrt[4]{\hat{\beta}}} = -\frac{\Omega}{\varepsilon S_1^2}\frac{1}{\sqrt{\hat{\beta}}} \varepsilon E_{max} S_1 \sqrt[4]{\hat{\beta}} + \alpha \tag{S15}$$

$$\frac{2\Omega E_{max}}{S_1 \sqrt[4]{\hat{\beta}}} = \alpha, \tag{S16}$$

leading to the relation between charge and voltage for the segment (C)-(D):

$$V = -\frac{\Omega}{\varepsilon S_1^2}\frac{1}{\sqrt{\hat{\beta}}} Q + \frac{2\Omega E_{max}}{S_1 \sqrt[4]{\hat{\beta}}} \tag{S17}$$

With respect to figure 3 of the main manuscript, we need to calculate the intersections of the line describing segment (C)-(D) with the isocapacitance lines C_1 and $\hat{\beta}C_1$, which describe the charge and voltage present on the DEG in its relaxed state (point 1') and at its maximal deformation (point 2').

For point 1':

$$V_{1'} = \frac{Q_{1'}}{C_1} = \frac{Q_{1'}\Omega}{\varepsilon S_1^2} = -\frac{\Omega}{\varepsilon S_1^2}\frac{1}{\sqrt{\hat{\beta}}} Q_{1'} + \frac{2\Omega E_{max}}{S_1 \sqrt[4]{\hat{\beta}}}$$

$$\frac{Q_{1'}\Omega}{\varepsilon S_1^2}\left(1 + \frac{1}{\sqrt{\hat{\beta}}}\right) = \frac{2\Omega E_{max}}{S_1 \sqrt[4]{\hat{\beta}}}$$

$$Q_{1'} = \frac{2\varepsilon E_{max} S_1}{\hat{\beta}^{1/4} + \hat{\beta}^{-1/4}} \tag{S18}$$

$$V_{1'} = \frac{2E_{max}\Omega}{S_1\left(\hat{\beta}^{1/4} + \hat{\beta}^{-1/4}\right)}. \tag{S19}$$

For point 2':

$$V_{2'} = \frac{Q_{2'}}{\hat{\beta}C_1} = \frac{Q_{2'}\Omega}{\hat{\beta}\varepsilon S_1^2} = -\frac{\Omega}{\varepsilon S_1^2}\frac{1}{\sqrt{\hat{\beta}}}Q_{2'} + \frac{2\Omega E_{max}}{S_1\sqrt[4]{\hat{\beta}}}$$

$$\frac{Q_{2'}\Omega}{\varepsilon S_1^2}\left(\frac{1}{\hat{\beta}} + \frac{1}{\sqrt{\hat{\beta}}}\right) = \frac{2\Omega E_{max}}{S_1\sqrt[4]{\hat{\beta}}}$$

$$Q_{2'} = \frac{2\varepsilon E_{max}S_1}{\hat{\beta}^{-3/4} + \hat{\beta}^{-1/4}} \tag{S20}$$

$$V_{2'} = \frac{2E_{max}\Omega}{S_1\left(\hat{\beta}^{1/4} + \hat{\beta}^{3/4}\right)} \tag{S21}$$

The values of the voltage and electric charge at points 1' and 2' are summarised in table S2.

Point	V	Q
1'	$\frac{2E_{max}\Omega}{S_1\left(\hat{\beta}^{1/4} + \hat{\beta}^{-1/4}\right)}$	$\frac{2\varepsilon E_{max}S_1}{\hat{\beta}^{1/4} + \hat{\beta}^{-1/4}}$
2'	$\frac{2E_{max}\Omega}{S_1\left(\hat{\beta}^{1/4} + \hat{\beta}^{3/4}\right)}$	$\frac{2\varepsilon E_{max}S_1}{\hat{\beta}^{-3/4} + \hat{\beta}^{-1/4}}$

Table S2. Voltage and charge values at points 1' and 2' (main manuscript figure 3) for the OT cycle.

The value of $V_{2'}$ defines the voltage at which the charging of the DEG must be stopped, and the charge and voltage values at points 1' and 2' are used to calculate the priming input energy and the output energy, in order to quantify the net energy gain per cycle. A demonstration that this cycle does indeed correspond to the optimal triangular cycles contained into the feasible space is given in section 3.5.

3 NON-OPTIMAL TRIANGLE CYCLE

We analyse the quantity of harvested energy if the storage capacitor has a value that is not necessarily the optimal value. We define the storage capacitor as $C_s = \gamma C_1$, which defines the slope of the relaxation process. To select the optimal value of the priming voltage, 3 different cases need to be considered (Fig S1).

1. $0 \leq \gamma < 1$ (magenta). When $\gamma = 1$, point 1' coincides with point 1. More energy can be harvested for smaller values of γ if the relaxation curve is pinched at point 1, rather than made tangent with the maximal electric field line.
2. $1 \leq \gamma \leq \hat{\beta}$ (green). Voltage $V_{2'}$ is chosen so that the relaxation segment 2'-1' becomes tangent with the maximal electric field line. This is the same situation as for the optimal cycle, but with the slope of the segment imposed by C_{s2}. Similar equations as presented in section 2 can be derived using γ as an additional input.

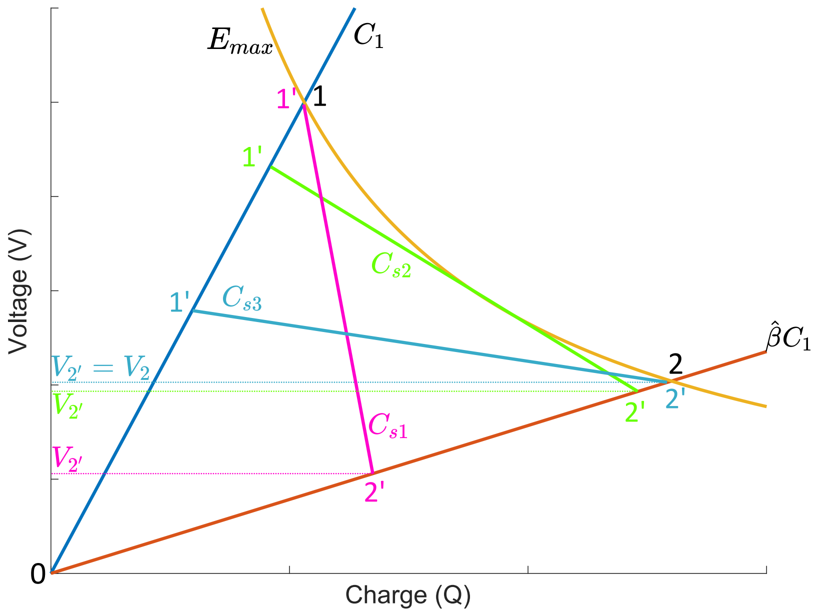

Figure S1. Different values of the storage capacitor C_s ($C_{s1} \le C_{s2} \le C_{s3}$) lead to different slopes of the relaxation line 2'-1'. The charging voltage $V_{2'}$ can then be chosen to make sure that the maximal field is reached at one point during the cycle, hence maximising the harvested energy

3. $\gamma > \hat{\beta}$ (turquoise). When $\gamma = \hat{\beta}$, point 2' coincides with point 2. More energy can be harvested for larger values of γ if the relaxation curve is pinched at point 2, rather than made tangent with the maximal electric field line.

The next sections establish the equations required to find the priming voltage V_2' and the voltage at the end of the relaxation V_1' for the three different cases. The values are then summarised in table S3.

3.1 $0 \le \gamma < 1$

We know that the relaxation line has a slope equal to $-1/Cs = -1/\gamma C_1$ and must go through point 1, i.e. through (Q_1, V_1). Consequently, the equation of the relaxation line is:

$$V = -\frac{Q}{\gamma C_1} + \alpha$$
$$V = -\frac{\Omega Q}{\gamma \varepsilon S_1^2} + \alpha$$

Forcing the line to go through the point (Q_1, V_1) (c.f. table S1) enables to identify the value of the intercept α.

$$V_1 = -\frac{\Omega Q_1}{\gamma \varepsilon S_1^2} + \alpha$$
$$\frac{\Omega E_{max}}{S_1} = -\frac{\Omega \varepsilon S_1 E_{max}}{\gamma \varepsilon S_1^2} + \alpha$$
$$\alpha = \frac{\Omega E_{max}}{S_1} \frac{\gamma + 1}{\gamma}$$

This leads to the following expression for the relaxation line (segment 2'-1'):

$$V = -\frac{\Omega Q}{\gamma \varepsilon S_1^2} + \frac{\Omega E_{max}}{S_1}\frac{\gamma+1}{\gamma} \tag{S22}$$

Point 2' defining the end of priming is obtained by calculating the intersection between the relaxation line and the isocapacitance line $\hat{\beta}C_1$: $V = Q\Omega/\hat{\beta}\varepsilon S_1^2$

$$\frac{Q_2'\Omega}{\hat{\beta}\varepsilon S_1^2} = -\frac{\Omega Q_2'}{\gamma\varepsilon S_1^2} + \frac{\Omega E_{max}}{S_1}\frac{\gamma+1}{\gamma}$$

$$Q_2' = \varepsilon E_{max} S_1 \frac{\gamma+1}{\gamma}\frac{\gamma\hat{\beta}}{\gamma+\hat{\beta}} \tag{S23}$$

$$V_2' = \frac{\Omega E_{max}}{S_1}\frac{\gamma+1}{\gamma+\hat{\beta}} \tag{S24}$$

For this case, the relaxation ends at point 1 (i.e. point 1'= point 1), with the values of voltage and charge given in table S1.

3.2 $1 \leq \gamma \leq \hat{\beta}$

This situation is very similar to the section 2, except that the slope of the relaxation line 2'-1' is defined by the value of the storage capacitor. First, we need to find the point (Q_{tg},V_{tg}) at which the relaxation line is tangent to the maximal field line, which happens when the slopes of the two lines are equal. The slope of the maximal electric field line is given by eq. S11. The slope of segment 2'-1' is given by:

$$\frac{dV}{dQ} = -\frac{1}{\gamma C_1} = -\frac{\Omega}{\gamma\varepsilon S_1^2} \tag{S25}$$

Equating Eqs. S11 and S25, and taking into account that the tangent point lies on the maximal field line described by eq. S7 enables to identify the point (Q_{tg},V_{tg}):

$$Q_{tg} = \sqrt{\gamma}\varepsilon E_{max} S_1 \tag{S26}$$

$$V_{tg} = \frac{\Omega E_{max}}{S_1\sqrt{\gamma}} \tag{S27}$$

The equation of segment 2'-1' can then be established using its slope (eq. S25):

$$V = -\frac{\Omega Q_1}{\gamma\varepsilon S_1^2} + \alpha \tag{S28}$$

The fact it needs to go through the point (Q_{tg},V_{tg}) is used to identify the intercept α:

$$\frac{\Omega E_{max}}{S_1\sqrt{\gamma}} = -\frac{\Omega\sqrt{\gamma}\varepsilon E_{max} S_1}{\gamma\varepsilon S_1^2} + \alpha$$

$$\alpha = \frac{2\Omega E_{max}}{\sqrt{\gamma}S_1}$$

And therefore, the equation of segment 2'-1' is given by:

$$V = -\frac{\Omega Q}{\gamma \varepsilon S_1^2} + \frac{2\Omega E_{max}}{\sqrt{\gamma} S_1} \tag{S29}$$

Point 1' reached at the end of the relaxation process is given by the intersection of segment (C)-(D) with the isocapacitance straight line with slope $1/C_1$:

$$\begin{aligned}
V =& -\frac{\Omega Q}{\gamma \varepsilon S_1^2} + \frac{2\Omega E_{max}}{\sqrt{\gamma} S_1} \\
\frac{Q_1'\Omega}{\varepsilon S_1^2} =& -\frac{\Omega Q_1'}{\gamma \varepsilon S_1^2} + \frac{2\Omega E_{max}}{\sqrt{\gamma} S_1} \\
Q_1' =& 2\varepsilon S_1 E_{max} \frac{\sqrt{\gamma}}{\gamma + 1} \qquad \text{(S30)} \\
V_1' =& \frac{Q_1'}{C_1} = \frac{\Omega E_{max}}{S_1} \frac{2\sqrt{\gamma}}{\gamma + 1} \qquad \text{(S31)}
\end{aligned}$$

Point 2' reached at the end of the priming phase is given by the intersection of segment (C)-(D) with the isocapacitance straight line with slope $1/\hat{\beta} C_1$:

$$\begin{aligned}
V =& -\frac{\Omega Q}{\gamma \varepsilon S_1^2} + \frac{2\Omega E_{max}}{\sqrt{\gamma} S_1} \\
\frac{Q_2'\Omega}{\hat{\beta} \varepsilon S_1^2} =& -\frac{\Omega Q_2'}{\gamma \varepsilon S_1^2} + \frac{2\Omega E_{max}}{\sqrt{\gamma} S_1} \\
Q_2' =& 2\varepsilon S_1 E_{max} \frac{\sqrt{\gamma}\hat{\beta}}{\gamma + \hat{\beta}} \qquad \text{(S32)} \\
V_2' =& \frac{Q_1'}{\hat{\beta} C_1} = \frac{\Omega E_{max}}{S_1} \frac{2\sqrt{\gamma}}{\hat{\beta} + \gamma} \qquad \text{(S33)}
\end{aligned}$$

3.3 $\gamma > \hat{\beta}$

We know that the relaxation line has a slope equal to $-1/Cs = -1/\gamma C_1$ and must go through point 2, i.e. through (Q_2, V_2). Consequently, the equation of the relaxation line is:

$$\begin{aligned}
V =& -\frac{Q}{\gamma C_1} + \alpha \\
V =& -\frac{\Omega Q}{\gamma \varepsilon S_1^2} + \alpha
\end{aligned}$$

Forcing the line to go through the point (Q_2, V_2) (c.f. table S1) enables to identify the value of the intercept α.

$$
\begin{aligned}
V_2 &= -\frac{\Omega Q_2}{\gamma \varepsilon S_1^2} + \alpha \\
\frac{\Omega E_{max}}{\sqrt{\hat{\beta}} S_1} &= -\frac{\Omega \varepsilon S_1 E_{max} \sqrt{\hat{\beta}}}{\gamma \varepsilon S_1^2} + \alpha \\
\alpha &= \frac{\Omega E_{max}}{S_1} \left(\frac{\sqrt{\hat{\beta}}}{\gamma} + \frac{1}{\sqrt{\hat{\beta}}} \right)
\end{aligned}
$$

This leads to the following expression for the relaxation line (segment 2'-1'):

$$
V = -\frac{\Omega Q}{\gamma \varepsilon S_1^2} + \frac{\Omega E_{max}}{S_1} \frac{\gamma + \hat{\beta}}{\gamma \sqrt{\hat{\beta}}} \tag{S34}
$$

For this case, the relaxation starts at point 2 (i.e. point 2'= point 2), with the values of voltage and charge given in table S1. Point 1' defining the end of relaxation is obtained by calculating the intersection between the relaxation line and the isocapacitance line C_1: $V = Q\Omega / \varepsilon S_1^2$

$$
\frac{Q_1' \Omega}{\varepsilon S_1^2} = -\frac{\Omega Q_1'}{\gamma \varepsilon S_1^2} + \frac{\Omega E_{max}}{S_1} \frac{\gamma + \hat{\beta}}{\gamma \sqrt{\hat{\beta}}}
$$

$$
Q_1' = \varepsilon E_{max} S_1 \frac{\gamma + \hat{\beta}}{\sqrt{\hat{\beta}} (\gamma + 1)} \tag{S35}
$$

$$
V_1' = \frac{\Omega E_{max}}{S_1} \frac{\gamma + \hat{\beta}}{\sqrt{\hat{\beta}} (\gamma + 1)} \tag{S36}
$$

3.4 Summary and energy density

Table S3 shows the voltage at the beginning ($V_{2'}$) and at the end ($V_{1'}$) of the relaxation part of the cycle during which voltage boosting occurs, summarising the calculations from the three previous sections. It can be verified that by using $\gamma = \sqrt{\hat{\beta}}$ the values become identical to those for the optimal triangle cycle (Eqs. S19 and S21). The values from the table can then be used to calculate the priming energy W_{in}, the stored energy W_{out}, and the net energy density generated per cycle w:

	$V_{1'}$	$V_{2'}$
$0 \leq \gamma < 1$	$\frac{\Omega E_{max}}{S_1}$	$\frac{\Omega E_{max}}{S_1}\frac{\gamma+1}{\gamma+\hat{\beta}}$
$1 \leq \gamma \leq \hat{\beta}$	$\frac{\Omega E_{max}}{S_1}\frac{2\sqrt{\gamma}}{\gamma+1}$	$\frac{\Omega E_{max}}{S_1}\frac{2\sqrt{\gamma}}{\hat{\beta}+\gamma}$
$\gamma > \hat{\beta}$	$\frac{\Omega E_{max}}{S_1}\frac{\gamma+\hat{\beta}}{\sqrt{\hat{\beta}}\,(\gamma+1)}$	$\frac{\Omega E_{max}}{\sqrt{\hat{\beta}}S_1}$

Table S3. Voltage value at the end ($V_{1'}$) and at the beginning ($V_{2'}$) of the relaxation phase, as a function of the capacitance swing $\hat{\beta}$, and the relative capacitance of the storage capacitor γ.

$$W_{in} = \frac{1}{2}\left(\hat{\beta}C_1 + C_s\right)V_{2'}^2 = \frac{\varepsilon S_1^2}{2\Omega}\left(\hat{\beta}+\gamma\right)V_{2'}^2 \tag{S37}$$

$$W_{out} = \frac{1}{2}\left(C_1 + C_s\right)V_{1'}^2 = \frac{\varepsilon S_1^2}{2\Omega}\left(1+\gamma\right)V_{1'}^2 \tag{S38}$$

$$w = \frac{W_{out} - W_{in}}{\Omega} = \frac{\varepsilon S_1^2}{2\Omega^2}\left[\left(1+\gamma\right)V_{1'}^2 - \left(\hat{\beta}+\gamma\right)V_{2'}^2\right] \tag{S39}$$

3.5 Proof that the OT cycle represents the optimal cycle

In section 2, we have established the equations of a triangular cycle whose discharge slope was defined by the line linking the intersection of the maximal field line with the two isocapacitance lines (Figure 3 of the main manuscript). We have called this cycle the optimal cycle, but without demonstrating that it maximises the harvested energy density. The equations developed in section 3 enables us to show that it is indeed the case.

Figure S1 shows that the surface of the triangle is maximised when the relaxation (segment 2'-1') is tangent with the maximal field line. This is the case C_{s2} on the figure, i.e. when $1 \leq \gamma \leq \hat{\beta}$. The harvested energy density is given by eq. S39. The values of $V_{1'}$ and $V_{2'}$ are given in the second row of table S3. Introducing these values in eq. S39 leads to:

$$w \propto \left[\frac{(1+\gamma)\,4\gamma}{(1+\gamma)^2} - \frac{\left(\hat{\beta}+\gamma\right)4\gamma}{\left(\hat{\beta}+\gamma\right)^2}\right]$$

$$w \propto \left[\frac{4\gamma}{(1+\gamma)} - \frac{4\gamma}{\left(\hat{\beta}+\gamma\right)}\right]$$

We can then differentiate with respect to γ and find the maximum or minimum of the function:

$$\frac{\partial w}{\partial \gamma} \propto \left[\frac{4}{(1+\gamma)^2} - \frac{4\hat{\beta}}{\left(\hat{\beta}+\gamma\right)^2} \right] = 0$$

$$\begin{aligned} \frac{4}{(1+\gamma)^2} =& \frac{4\hat{\beta}}{\left(\hat{\beta}+\gamma\right)^2} \\ (1+\gamma)^2 =& \frac{\left(\hat{\beta}+\gamma\right)^2}{\hat{\beta}} \\ \hat{\beta} + 2\hat{\beta}\gamma + \hat{\beta}\gamma^2 =& \gamma^2 + 2\hat{\beta}\gamma + \hat{\beta}^2 \\ (\hat{\beta}-1)\gamma^2 =& \hat{\beta}(\hat{\beta}-1) \\ \gamma^2 =& \hat{\beta} \end{aligned}$$

As γ must be positive, this leads to $\gamma = \sqrt{\hat{\beta}}$. The second derivative is negative at that point, which hence represents a local maximum. Therefore, the area of the harvested triangle is maximised for $C_s = \sqrt{\hat{\beta}}C_1$, which corresponds to the value of eq. S10.

4 SET-AND-FORGET APPROACH

Figure S2 illustrates the set-and-forget concept in the Q-V plane. The circuit is designed for a set value of the capacitance swing $\hat{\beta}_s$. This defines the storage capacitance C_s and therefore the slope of segment 1'-2', as well as the priming voltage $V_{2'}$ according to the equations of the optimal triangle cycle (OT). The obtained cycle is shown in purple. The cycle caused by a deformation amplitude $\hat{\beta} < \hat{\beta}_s$ is illustrated in green. As the priming voltage $V_{2'}$ is fixed, the priming stops at point 2" and the discharge slope (2"-1") is identical to that of segment (2'-1') due to the fixed value of the storage capacitor. The green triangle illustrates the energy harvested in this condition, which is much lower than the maximal harvestable energy for a capacitance swing $\hat{\beta}$; the maximal electric field is never reached during the cycle. The figure also shows in gray dashed line the outline of a cycle with a capacitance swing $\hat{\beta} > \hat{\beta}_s$. In that case, the maximal electric field E_{max} is exceeded during the cycle, which must be avoided. Consequently, the harvester must be designed so that $\hat{\beta} \leq \hat{\beta}_s$, which can be done by the implementation of a mechanical stop that limits the deformation of the harvester. Instead of a mechanical stop, the setting point of the circuit $\hat{\beta}_s$ could be chosen sufficiently high to ensure that the electric field in the device remains lower than E_{max}. For a normal distribution of $\hat{\beta}$, with a mean value μ and a standard deviation σ, choosing $\hat{\beta}_s = \mu + 3\sigma$ would ensure that $99.87\,\%$ of the cycles respect the electric field criteria. However, choosing $\hat{\beta}_s$ significantly higher than the mean value of the normal distribution leads to a sub-optimal amount of harvested energy (see fig 6 b of the main article), and consequently, a mechanical stop is the option that should be implemented.

We now calculate the energy harvested per cycle for a capacitance swing $\hat{\beta}$ when the harvesting circuit is optimised for a capacitance swing $\hat{\beta}_s$, which is illustrated by the green triangle on Figure S2. The voltage $V_{2'}$ is that of the OT cycle and given in table S2, when calculated for a capacitance swing of $\hat{\beta}_s$, and

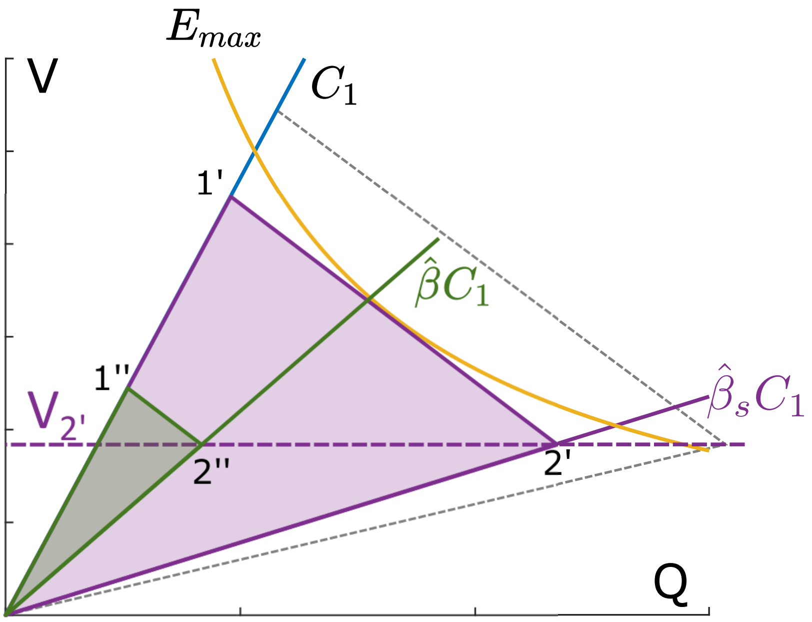

Figure S2. Illustration of the set-and-forget-approach on the Q-V plane. The purple triangle illustrates the OT cycle for a target deformation $\hat{\beta}_s$ that is used to select the storage capacitance and priming voltage. The green cycle illustrates the energy harvested for a capacitance swing $\hat{\beta} < \hat{\beta}_s$, while the gray dashed lines outline a cycle for $\hat{\beta} > \hat{\beta}_s$, that must be avoided as the electric field E_{max} is exceeded.

$V_{2''} = V_{2'}$. Consequently, the charge at point $Q_{2''}$ can be calculated as:

$$Q_{2''} = \hat{\beta} C_1 V_{2''} = \varepsilon E_{max} S_1 \frac{2\hat{\beta}}{\hat{\beta}_s^{1/4} + \hat{\beta}_s^{3/4}} \tag{S40}$$

Segment 1”-2” has the same slope as segment 1’-2’ (Eq. S9), and the equation of segment 1”-2” is consequently given by:

$$V = -\frac{\Omega}{\varepsilon S_1^2 \sqrt{\hat{\beta}_s}} Q + \alpha. \tag{S41}$$

As we know that the line goes through the point $Q_{2''}$ and $V_{2''}$, we can calculate the value of the offset α and write the equation of the discharge line 1”-2”:

$$V = -\frac{\Omega}{\varepsilon S_1^2 \sqrt{\hat{\beta}_s}} Q + \frac{\Omega E_{max}}{S_1} \frac{2\left(\sqrt{\hat{\beta}_s} + \hat{\beta}\right)}{\hat{\beta}_s^{5/4} + \hat{\beta}_s^{3/4}}. \tag{S42}$$

It can be verified that when $\hat{\beta} = \hat{\beta}_s$, the equation of line 1”-2” becomes that of line 1’-2’ given by eq. S17. With this equation, we can calculate the coordinates of point 1” which is the intersection with the isocapacitance line C_1:

$$V_{1''} = \frac{Q_{1''}}{C_1} = \frac{Q_{1''}\Omega}{\varepsilon S_1^2} = -\frac{\Omega}{\varepsilon S_1^2\sqrt{\hat{\beta}_s}} Q_{1''} + \frac{\Omega E_{max}}{S_1}\frac{2\left(\sqrt{\hat{\beta}_s}+\hat{\beta}\right)}{\hat{\beta}_s^{5/4}+\hat{\beta}_s^{3/4}}$$

$$\frac{Q_{1''}\Omega}{\varepsilon S_1^2}\frac{\sqrt{\hat{\beta}_s}+1}{\sqrt{\hat{\beta}_s}} = \frac{\Omega E_{max}}{S_1}\frac{2\left(\sqrt{\hat{\beta}_s}+\hat{\beta}\right)}{\hat{\beta}_s^{5/4}+\hat{\beta}_s^{3/4}}$$

$$Q_{1'} = \varepsilon E_{max} S_1 \frac{2\left(\sqrt{\hat{\beta}_s}+\hat{\beta}\right)}{\left(\sqrt{\hat{\beta}_s}+1\right)^2\sqrt[4]{\hat{\beta}_s}} \tag{S43}$$

$$V_{1'} = \frac{E_{max}\Omega}{S_1}\frac{2\left(\sqrt{\hat{\beta}_s}+\hat{\beta}\right)}{\left(\sqrt{\hat{\beta}_s}+1\right)^2\sqrt[4]{\hat{\beta}_s}}. \tag{S44}$$

The values of voltage and charge at points 1" and 2" can then be used to calculate the energy invested for priming and harvested during charge collection, as well as the resulting net energy density generated per cycle:

$$W_{in} = \frac{1}{2}\left(\hat{\beta}C_1 + C_s\right)V_{2'}^2 = \frac{\varepsilon S_1^2}{2\Omega}\left(\hat{\beta}+\sqrt{\hat{\beta}_s}\right)V_{2''}^2 \tag{S45}$$

$$W_{out} = \frac{1}{2}\left(C_1 + C_s\right)V_{1'}^2 = \frac{\varepsilon S_1^2}{2\Omega}\left(1+\sqrt{\hat{\beta}_s}\right)V_{1''}^2 \tag{S46}$$

$$w = \frac{W_{out}-W_{in}}{\Omega} = \varepsilon E_{max}^2 \frac{2\left(\hat{\beta}-1\right)\left(\sqrt{\hat{\beta}_s}+\hat{\beta}\right)}{\left(\sqrt{\hat{\beta}_s}+1\right)^3\sqrt{\hat{\beta}_s}}. \tag{S47}$$

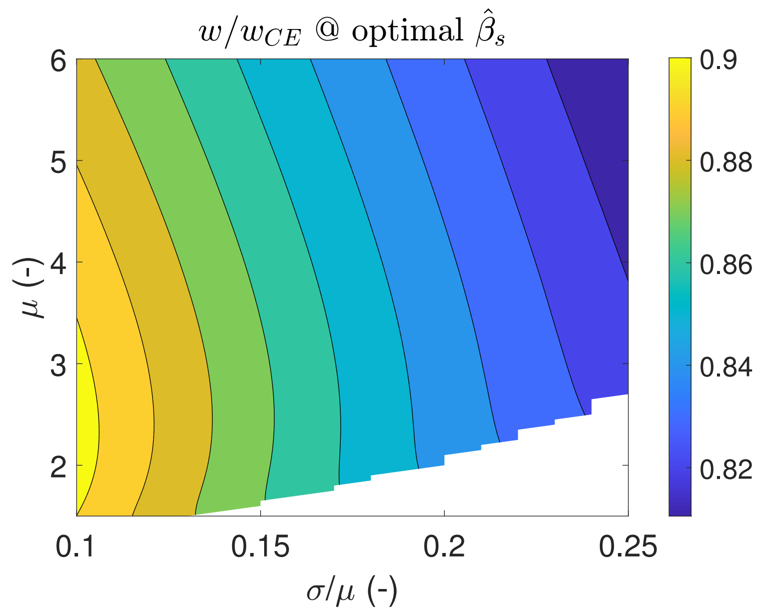

Figure S3. Normalised (with respect to the constant field cycle) average energy density output of the set and forget cycle for different parameters of the normal distribution of input capacitance swing (mean value μ and standard deviation σ. This assumes that the set point of the circuit has been optimised to the parameters of the normal distribution.

It can be verified that when $\hat{\beta} = \hat{\beta}_s$, the eq. S47 is equivalent to the energy density of the OT cycle (Eq. 5 from the main manuscript). These equations can then be used to calculate the generated energy for an energy input following a given statistical distribution. Here we consider a normal distribution of capacitance swing (see the main article). Provided that the circuit set point is chosen at the optimal value for the input distribution, then the amount of harvested energy can be quite high compared to the ideal constant electric field (CE) cycle (Figure S3). For the parameter space considered here, it is comprised between $81\,\%$ and $90\,\%$ of the maximal harvestable energy amount. Of course, this assumes that the parameters of the input distribution are known, so that the circuit can be tuned with the right value of $\hat{\beta}_s$.

5 EXPERIMENTAL VERIFICATION

An LCR meter (Hioki IM3523) was used to establish the relation between the displacement of the central hub and the capacitance. Five ramps between $10\,\mathrm{mm}$ and $70\,\mathrm{mm}$ and back were performed at $2\,\mathrm{mm\,s^{-1}}$ (Figure S4). The DEG membrane is not prestretched and has a bit of slack. It requires an initial offset to become taut. An analytical model of the form

$$\beta = \left(\frac{x^2 + \Delta R^2}{x_0^2 + \Delta R^2}\right)^n \tag{S48}$$

is fitted to the experimental capacitance swing data to model the relationship between the physical displacement of the membrane and capacitance swing β. x is the position of the hub with respect to the neutral position, ΔR is the difference between the membrane outer radius and the hub radius, x_0 is the displacement offset required to keep the membrane taut, and n accounts for non-linearity. If the DEG deforms like a cone as illustrated on the inset of figure S4, the value of parameter n should be $n = 1$. However, stretching the membrane in the radial direction induces a hoop stress, which affects the shape of

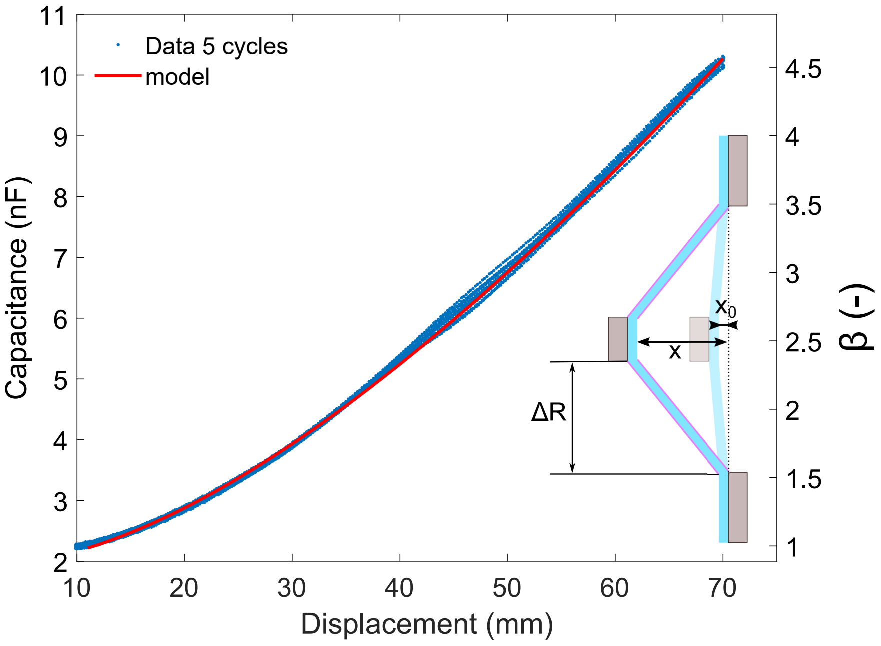

Figure S4. Measured capacitance of the DEG vs. central hub position for five ramps (blue) and fitted model (red). The corresponding capacitance swing is indicated in the right axis. Inset: parameter of the model

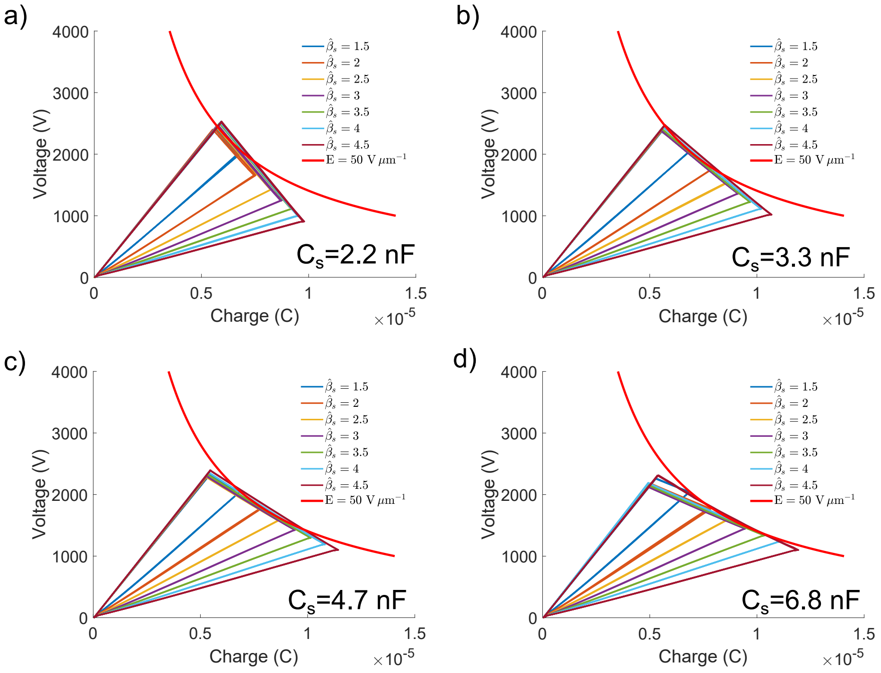

Figure S5. Harvesting cycles for capacitance swings between 1.5 and 4.5 and different values of storage capacitor

the membrane, as visible in figure 8 of the main manuscript. A fit on the experimental data leads to the following values: $\Delta R = 22.6$ mm, $x_0 = 11.6$ mm and $n = 0.72$ (R^2 value: 0.9992).

Figure S5 shows the harvesting cycles between $\hat{\beta} = 1.5$ to $\hat{\beta} = 4.5$. 5 cycles are performed and displayed on the graph for each value of $\hat{\beta} = 1.5$. For each group of 5 cycles, the collected energy (surface of the

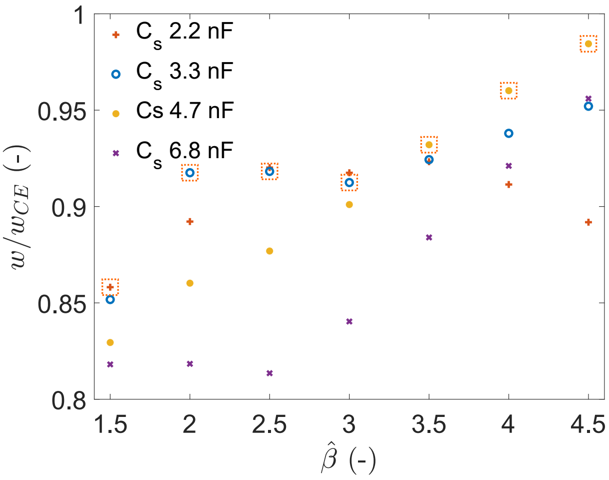

Figure S6. harvested energy density for each of the cycles of figure S5, relative to the harvestable energy as defined by the constant field (CE) cycle. For each value of $\hat{\beta}$, the relative energy density for the capacitor value that is the closes match to the optimal value is indicated with a dotted red frame.

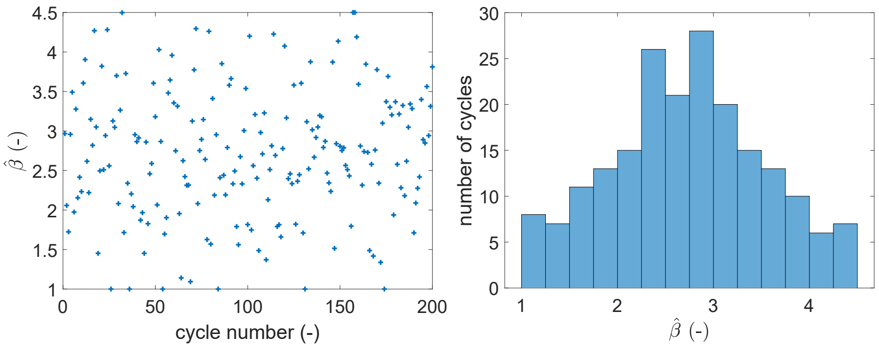

Figure S7. Left: 200 random values of capacitance swing following a normal distribution with parameters $\mu = 2.75$ and $\sigma = 0.8$. Right: histogram of the distribution.

triangle)is calculated and averaged. The energy density (both absolute and normalise) is shown in figure 9d of the main article. Figure S6 shows the energy density of each combination of deformation and storage capacitor relative to that of the constant field cycle. The constant field cycles represents the largest amount of energy that can be collected without exceeding the defined maximal electric field, and figure S6 therefore shows the fraction of harvestable energy that is effectively collected by the electronic circuit. Each value of capacitance swing has an optimal value of capacitance swing. On S6, we have highlighted with a red frame, the energy density corresponding to a capacitor value that is the closes to the optimal value. In the range of tested capacitance swing, and with the most appropriate storage capacitor, the fraction of collected energy is in the range of $86\,\%$ to $98\,\%$.

To test a distribution of capacitance swing, a normal distribution is used, with a mean $\mu = 2.75$ and a standard deviation $\sigma = 0.8$. 200 capacitance swing values $\hat{\beta}$ are then generated according to this distribution. Figure S7 shows one of the distributions used for testing. Each test is made with a new random collection of 200 capacitance swing values, following the same normal distribution.

Figure S8 shows the generated energy density for the 200 normally-distributed capacitance swing values, for 4 values of storage capacitors.

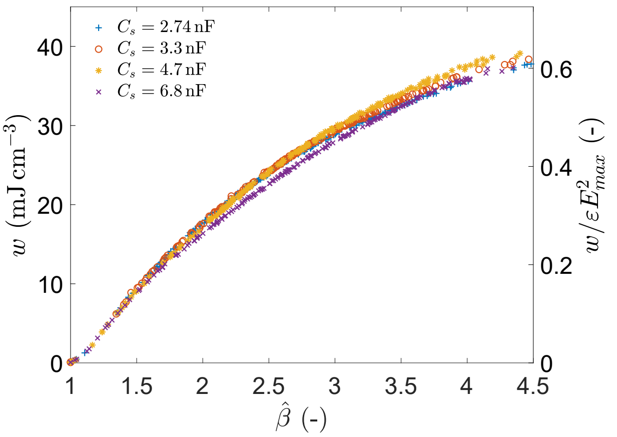

Figure S8. Energy density (left axis) and normalised energy density (right axis) generated for 200 cycles normally distributes with parameters$\mu = 2.75$ and $\sigma = 0.8$, for 4 different values of storage capacitor C_s.
